# Gram-positive probiotics improves acetaminophen-induced hepatotoxicity by inhibiting leucine and Hippo-YAP pathway

**DOI:** 10.1186/s13578-025-01370-5

**Published:** 2025-03-07

**Authors:** Wenkang Gao, Gang Wang, Hang Yuan, Yue Chen, Jiake Che, Zilu Cheng, Liuying Chen, Li Zhang, Yuanqing Zhu, Xin Liu, Ao Liu, Quancheng Yang, Peng Cao, Wei Qian, Weiyan Huang, Bernd Schnabl, Ling Yang, Huikuan Chu

**Affiliations:** 1 Division of Gastroenterology, Union Hospital, Tongji Medical College, Huazhong University of Science and Technology, 1277 Jiefang Avenue, Wuhan, 430022 Hubei China; 2 Wuhan Mental Health Center, Wuhan, 430022 Hubei China; 3 Department of Respiratory Medicine, the 904 Hospital of the Joint Logistics Support Force of the Chinese People’S Liberation Army, Wuxi, Jiangsu, 214000 China; 4 School of Basic Medicine, Tongji Medical College, Huazhong University of Science and Technology, Wuhan, China; 5 Department of Pharmacy, Union Hospital, Tongji Medical College, Huazhong University of Science and Technology, Wuhan, 430022 China; 6 Department of Medicine, University of California, San Diego, 9500 Gilman Drive, La Jolla, CA MC0063 USA

**Keywords:** Acetaminophen, Dysbiosis, Probiotics, Gut-liver axis, Branched-chain amino acids, Gut microbiome

## Abstract

**Objectives:**

Drug-induced liver injury (DILI) can be improved by modulating gut microbiota. We aimed to investigate a probiotic mixture comprising *Bifidobacterium Longum*, *Streptococcus thermophilus*, and *Lactobacillus delbrueckii subspecies bulgaricus* (BSL) in mitigating acetaminophen induced liver injury (AILI), and to elucidate the underlying mechanisms.

**Methods:**

Gut bacterial communities were analyzed in fecal samples from patients with DILI and healthy controls. Mice were pretreated with BSL or PBS for 10 days, then subjected to a single dose of acetaminophen (300 mg/kg) gavage and euthanized 24 h later. Transcriptome sequencing, microbiome, and metabolome sequencing were performed on mouse samples, respectively.

**Results:**

Gut bacterial dysbiosis existed in DILI patients, with a decrease in Gram-positive bacteria and an increase in Gram-negative bacteria. A similar situation occurred in AILI mice. Pretreatment of BSL significantly improved APAP-induced disorders of gut bacteria and alleviated hepatic inflammation and necrosis. Transcriptome sequencing showed that BSL inhibited the hepatic damage pathways, such as Hippo and TGF-β signaling pathway. Metabolomic profiling revealed an obvious increase in oligopeptides containing branched-chain amino acids (BCAAs) in AILI mice, whereas these metabolites were significantly negatively correlated with the abundance of BSL, but positively with key genes of Hippo pathway. In vitro experiments showed that leucine exerted a dose-related exacerbation pattern on APAP-mediated hepatocellular injury. Mice supplemented with leucine resulted in the further overexpression of Yes-associated protein, an increase in oxidative stress, and a worsening of AILI.

**Graphical Abstract:**

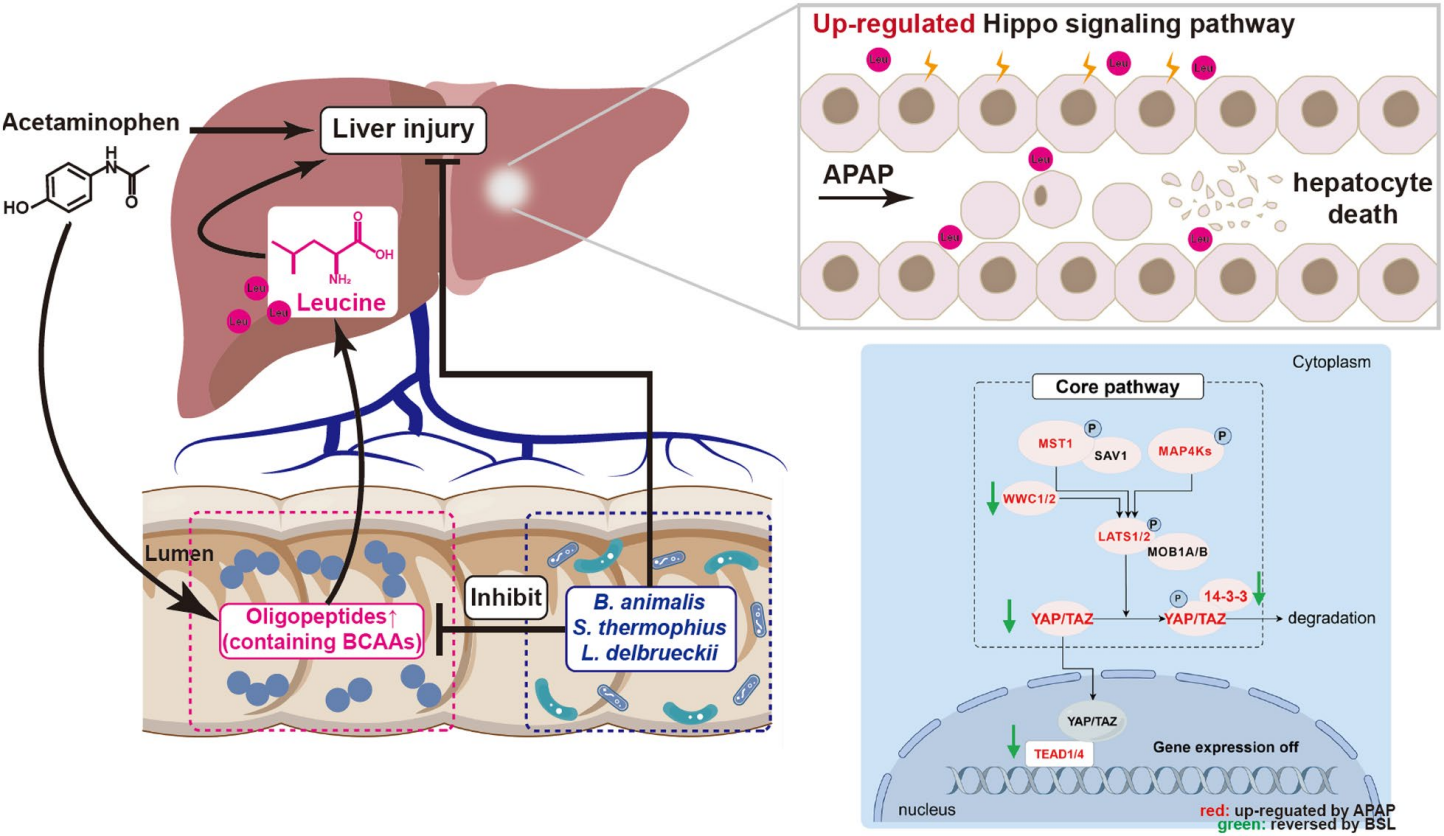

**Supplementary Information:**

The online version contains supplementary material available at 10.1186/s13578-025-01370-5.

## Introduction

Acetaminophen (APAP) is a classic and clinically widely used antipyretic and analgesic drug [1]. About 23% of adults (nearly 52 million people) in America use APAP-containing medications weekly [2]. Approximately 50% of acute liver failure (ALF) cases are caused by APAP and other drugs [3]. Given the small difference between the efficacy and toxicity doses of APAP, which makes it susceptible to overdose and consequent liver and kidney injury, the healthcare burden associated with APAP is a matter of concern.

Although APAP-induced liver injury (AILI) is thought to be associated with direct hepatotoxicity of APAP, recent evidence suggests that intestinal events, such as gut microbiota dysbiosis, impaired barrier function, and activation of inflammatory or metabolic pathways, play an important role in the process of AILI [4]. Growing evidence indicates that acute or chronic liver injury is strongly associated with gut microorganisms, and the importance of gut-liver interaction has been increasingly emphasized [5]. For instance, patients who take long-term antibiotic usually suffer from intestinal dysbiosis and barrier dysfunction, and they are more susceptible to AILI, resulting in a significantly increased risk of ALF [6]. Similarly, ampicillin can exacerbate AILI by inducing gut microbiota imbalance [7]. APAP overdose increases intestinal permeability, and gut-derived pathogens and metabolites can translocate to the liver, which induces hepatic inflammatory reactions [4, 8].

Currently, there are limited studies on the mechanism of intestinal microecology in AILI. The degree of hepatic inflammation and necrosis in AILI can be attenuated through supplementation of probiotics or inhibition of harmful bacteria. For example, supplementation of *Lactobacillus vaginalis* and *Bifidobacterium longum* can alleviate APAP-induced hepatotoxicity [9, 10]. Phenylpropionic acid, a metabolite produced by gut microbiota, has also been shown to attenuate AILI [11].

A preparation containing three probiotics (BSL), including *Bifidobacterium Longum*, *Streptococcus thermophilus*, and *Lactobacillus delbrueckii subspecies bulgaricus*, is commonly used clinically for the treatment of diarrhea resulting from intestinal microbiota disturbance. Prior studies supported its effects in ameliorating ethanol-induced acute liver injury in rats [12] and enhancing intestinal defenses to prevent translocation of harmful bacteria [13]. However, there is a paucity of research on the influence of gut microbes or the gut-derived metabolites on host metabolism, especially the pathway of the essential amino acids. In this study, we observed the protective role of BSL in AILI mice and explored the potential mechanisms from the perspective of branched-chain amino acids (BCAAs, including leucine, isoleucine, and valine) metabolism.

## Method and materials

### Patient selection and clinical trial design

To determine whether gut microbiota was altered in DILI patients, we conducted a single-center, prospective, observational study at Wuhan Union Hospital. The patients meeting all the following inclusion criteria were included: (1) aged > 18 years; (2) met the diagnostic criteria of DILI in Guidelines for Diagnosis and Treatment of DILI [14]; (3) with histories of using APAP; (4) with relatively complete clinical data and good compliance. Patients were excluded according to the following exclusion criteria: (1) with hepatocellular carcinoma or hepatic metastases; (2) combined with infectious liver diseases, such as hepatitis A virus, hepatitis B virus, hepatitis C virus, hepatitis D virus, hepatitis E virus, and human immunodeficiency virus; (3) combined with non-infectious liver diseases, such as non-alcoholic fatty liver disease, alcoholic liver disease, autoimmune liver disease, immunoglobulin G4-related liver disease, Wilson's disease, alpha 1-antitrypsin deficiency, Budd-Chiari syndrome, and other congenital liver diseases; (4) combined with severe organic lesions; (5) pregnant and lactating women; (6) no antibiotics or proton pump inhibitors which affected gut microbiota in the past 1 month [15, 16]. The healthy control (HC) group met the following criteria: (1) aged > 18 years; (2) no history of liver disease or other organic diseases. All subjects involved in the study signed an informed consent agreement.

A total of 40 patients with liver diseases were recruited from Union Hospital of Huazhong University of Science and Technology from June 2022 to September 2023. After further screening, 16 patients and 18 healthy subjects were included in the DILI group and HC group, respectively (Supplementary Fig. 1A). Their whole blood specimens were centrifuged at 3000×*g* for 15 min at 4 ℃, plasma samples (upper layer) were then stored at −80℃ prior use. All subjects were instructed to collect their stool samples using sterile specimen containers and then to place them in a 4℃ refrigerator for temporary storage. Their fecal specimens were transported to the specimen bank of Union Hospital within 24 h for long-term storage at −80℃.

### Animals and treatment

C57BL/6J mice (male, weight: 20-25g, age: 7–8 weeks) housed in specific pathogen-free environment under a cycle of 12 h light and dark with food and water ad libitum. All mice received adaptive feeding for 7 days prior to the formal experiment. The mice were randomly divided as: APAP group, APAP_BSL group, BSL group, and control (CON) group. The groups of APAP_BSL and BSL were administered orally with 3 × 10^9 CFU BSL (provided by Inner Mongolia Shuang Qi Pharmaceutical Co., Ltd. *Bifidobacterium longum*: *Streptococcus thermophilus*: *Lactobacillus bulgaricus* = 1: 1: 1) suspended in 300 μl of sterile phosphate-buffered saline (PBS) each morning (8:00 am–10: 00 am), while those in groups of APAP and CON were gavaged with an equivalent dose of sterile PBS. The gavage treatment lasted for 10 days, and all the mice fasted for 12 h on the morning of day 9. In the evening of day 9, APAP groups were injected intraperitoneally with APAP (300 mg/kg), while those of CON and BSL were administered an equivalent volume of PBS as a control. All the mice were euthanized 24 h later.

To evaluate the effect of leucine on AILI, leucine (1.5% w/v) was added to the drinking water of APAP_Leu mice. APAP was used as above described. After 2 h of APAP injection, APAP_Leu group was gavaged with leucine (1.5% w/v, 10ml/kg) and APAP group was given an equivalent volume of PBS. All the mice were euthanized 22 h later.

To evaluate the role of the YAP inhibitor in AILI mice, Verteporfin (100mg/kg) was injected intraperitoneally before APAP injection, and then APAP was administrated 4 h later. All the mice were euthanized 24 h later.

### Ethics statement

The protocol of the clinical trial was registered at ClinicalTrials.gov (https://classic.clinicaltrials.gov) with registration number NCT05465642. The study was approved by the Ethics Committee of Union Hospital Affiliated to Tongji Medical College of Huazhong University of Science and Technology (Project number: UHCT22354, Ethics acceptance number: [2022]0383). This trial followed the Strengthening the Reporting of Observational Studies in Epidemiology (STROBE) reporting guideline and was conducted in compliance with Declaration of Helsinki. All patients informed consent and signed the consent form.

All experiments involving animals were approved by the Institutional Animal Ethics Committee of Huazhong University of Science and Technology. All animal experiments followed the Animal Research: Reporting of In Vivo Experiments (ARRIVE) guidelines.

### Serum and liver biochemical parameter analysis

Whole blood samples were kept overnight at 4℃, then centrifuged at 3000 × g for 15 min on the next day and reserved the supernatant for subsequent testing. The levels of serum alanine aminotransferase (ALT) and aspartate aminotransferase (AST) were measured using commercial assay kits (Jiancheng, Nanjing, China). Glutathione (GSH), oxidative glutathione (GSSG), malondialdehyde (MDA), and superoxide dismutase (SOD) were tested using appropriate kits (Jiancheng, Nanjing, China) according to the manufacturer’s instructions.

### Histopathological analysis

Liver tissues were stained with hematoxylin eosin to observe hepatic necrosis and inflammation. The percentage of liver necrotic area was calculated using NIH ImageJ software (imagej.net/ij).

### Cell culture and treatment

Human normal liver cells (L-02) were cultured in DMEM medium with 10% fetal bovine serum (FBS) (Gibco, USA). Then L-02 cells were starved with no FBS and incubated with APAP (TargetMol, Shanghai, China), leucine, isoleucine, and valine (Servicebio, Wuhan, China), respectively, for 24 h. Cell viability was detected by Cell Counting Kit 8 (HYCEZMBIO, Wuhan, China) following the manufacturer’s suggestions.

### Real-time quantitative PCR

RNA was isolated from mice liver using RNA easy isolation kit (Vazyme, Nanjing, China) and reversed transcribed to cDNA. Gene expression was determined with SYBR Green (Vazyme, Nanjing, China) using Roche Applied Science LightCycler 480 system. Primer sequences were obtained from PrimerBank (pga.mgh.harvard.edu/primerbank). All primers used in this paper are listed in Supplementary Table 4.

### Immunohistochemistry

The specific procedures were carried out as previously described [17]. Briefly, embedded liver sections were stained with cytochrome p450 1A2 (CYP1A2) and 2E1 (CYP2E1) antibodies following the standard procedure. The information of primary and secondary antibodies is shown in Supplementary Table 4.

### Western blot

Western blot was performed as described previously [17]. Primary antibodies, including anti-YAP1 (A19134, Abclonal, China), anti-phosphorylated YAP1 (AP0489, Abclonal, China), and anti-GAPDH (60,004–1-Ig, Proteintech) were used.

### Full-length 16S rRNA and ITS sequencing

For bacterial community analysis, the bacterial 16S rRNA genes were amplified using the broad-range bacterial primers 27F (5ʹ-AGRGTTYGATYMTGGCTCAG-3ʹ) and 1492R (5ʹ-RGYTACCTTGTTACGACTT-3ʹ) [18]. For fungal community analysis, the primers of ITS1F (5ʹ-CTTGGTCATTTAGAGGAAGTAA-3ʹ) and ITS4R (5ʹ-TCCTCCGCTTATTGATATGC-3ʹ) amplified the ITS sequences [19]. PCR amplification, libraries construction and sequencing were conducted at Majorbio Bio-Pharm Technology Co. Ltd. (Shanghai, China). The taxonomy of each OTU was analyzed by RDP Classifier version 2.13 against the 16S rRNA gene database (nt_v20221012/16s_bacteria) and ITS database (unite8.0/its_fungi) using confidence threshold of 0.7 [20]. The alpha-diversity was measured using the Sob, Shannon and Chao indices. The beta-diversity was evaluated by principal coordinate analysis (PCoA). The redundancy analysis (RDA) was performed to investigate the effect of clinical parameters on gut bacterial community structure. The linear discriminant analysis effect size (LEfSe) was used to identify differential species. The predicted metagenomic function of gut bacteria were performed using Phylogenetic Investigation of Communities by Reconstruction of Unobserved States (PICRUSt2) [21].

### Liquid chromatography-MS/MS

50 mg of mice cecal content was mixed with 400 μl extraction solution (methanol:water = 1:1 (v:v)), then it was grounded for 6 min (−10 ℃, 50 Hz). The mixture was centrifuged at 13,000×*g* for 15 min, and the supernatant was transferred to the injection vial for LC–MS/MS analysis. A pooled quality control sample (QC) was prepared by mixing all samples in equal volumes. The data were collected using a Thermo UHPLC-Q Exactive HF-X Mass Spectrometer according to the operating instructions. The raw data was analyzed by Progenesis QI 2.3 (Water Corporation, Milford, USA) software for peak detection. Internal standard peaks were deleted from data matrix. At least 80% of the metabolic features detected in any set of samples were retained. The maximum mass error allowed was ± 10 ppm, only the metabolites with MS/MS fragments score above 30 were considered as confidently identified. Partial Least Squares Discriminant Analysis (PLS-DA) was conducted to discriminate significant differences between the groups. Metabolites with consistent trends in each comparison group were used for subsequent analyses.

### RNA sequencing

Total RNA was extracted using the Trizol (Invitrogen, CA, USA). RNA purity and integrity were monitored by NanoDrop 2000 spectrophotometer (NanoDrop Technologies, Wilmington, DE, USA) and a Bioanalyzer 2100 system (Agilent Technologies, CA, USA). RNA was further purified using Oligo(dT)-attached magnetic beads. Purified mRNA was fragmented into small pieces with fragment buffer and reversed into cDNA. The cDNA fragments were amplified by PCR, and Ampure XP bead was used to purify the products and constructed the final library. HISAT2 (V2.1.0) was used to compare clean reads with the reference genome [22]. The expression levels were further quantified as Fragments Per Kilobase Per Million bases (FPKM) values using RSEM (V1.3.1) [23]. Differential expressed mRNAs were identified using the following criteria: fold change > 2 or < 0.5, and false discovery rate < 0.05. The signaling pathway was drawn using Figdraw 2.0 (www.figdraw.com).

### Statistical analysis

All data were expressed as mean ± standard error of the mean (SEM) unless otherwise specified. In mouse and cell culture experiments, one-way ANOVA test and Tukey’s post-hoc test were used for comparisons of > 2 groups within one experimental factor, two-way ANOVA test and Tukey’s post-hoc test for comparisons of > 2 groups within two experimental factors. The 16S rRNA, ITS, and metabolomics analysis were performed using the online platform of Majorbio Cloud (Majorbio Bio-Pharm Technology Co. Ltd., Shanghai, China, https://cloud.majorbio.com) [24] and R language (R Version 4.3.1).

## Results

### Patients with DILI showed gut dysbiosis

**Fig. 1:**
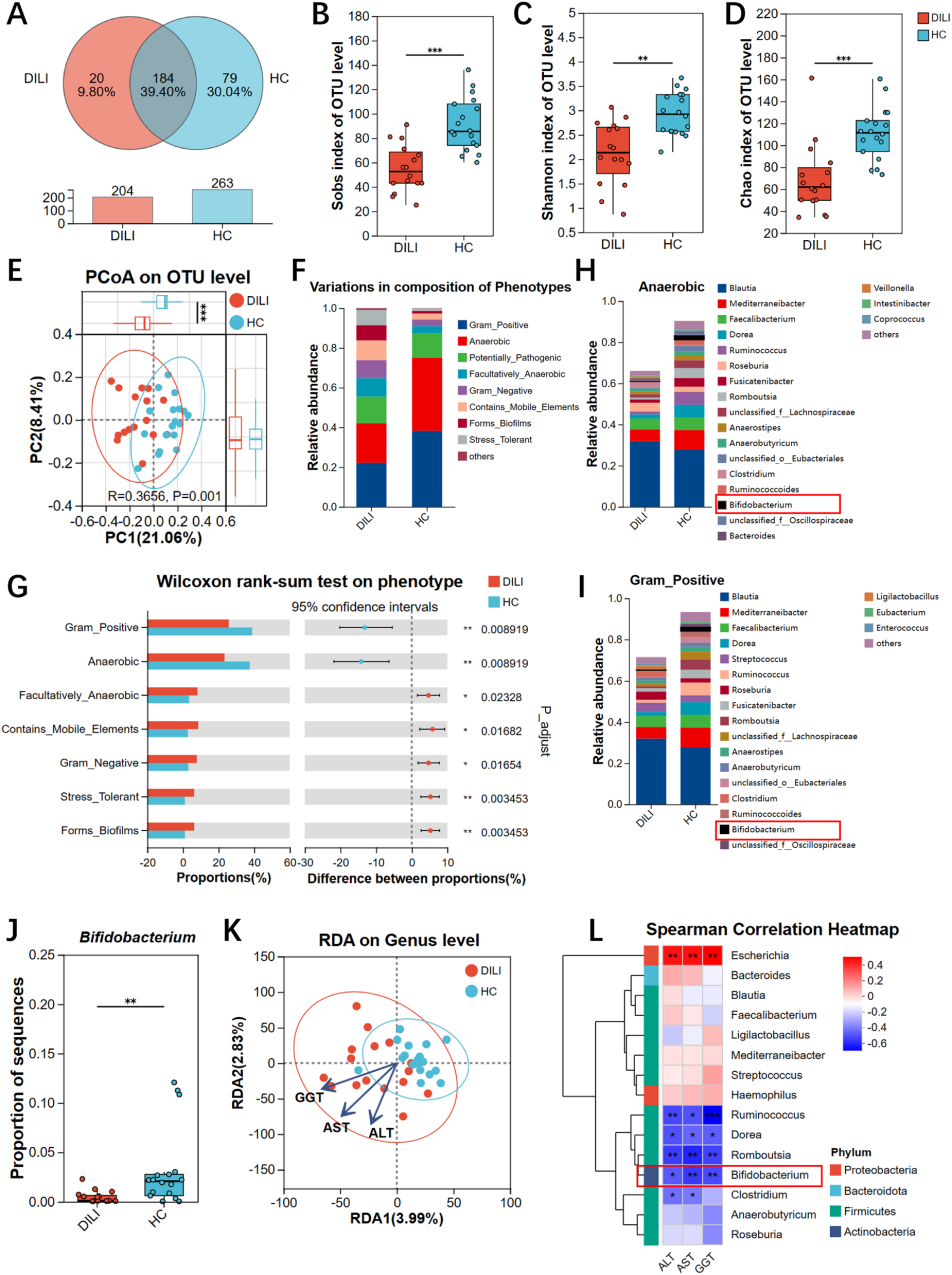
Patients with DILI showed gut dysbiosis. **A** Venn diagram of DILI group (n = 16) and HC group (n = 18) based on the OTU level of 16S rRNA sequencing. **B**-**D** The indices of Sobs, Shannon, and Chao at the OTU level of DILI and HC group (using Wilcoxon rank-sum test). **E** PcoA analysis of gut bacteria at the OTU level (using unweighted_unifrac distance algorithms, ANOSIM analysis compared the difference). **F** The prediction of phenotypic composition based on Bugbase phenotypic prediction (others indicated relative abundance less than 0.01). **G** Two-group difference test for predicted phenotypic composition (using Wilcoxon rank-sum test, p values were corrected by false discovery rate). **H**-**I** Bar plots of bacterial genera (ranked by relative abundance) contributing significantly to the corresponding phenotype. **J** The proportion of *Bifidobacterium* in two groups (using Wilcoxon rank-sum test). **K** The plot of redundancy analysis. The black blue arrows were the common clinical indicators of liver injury, *i.e.*, ALT, AST, and GGT. The longer the length of the arrow, the greater the influence of the indicator on the composition of gut microbiota. The red and blue dots represented the genera of DILI patients and healthy individuals, respectively. The closer the distance between the two points, the more similar the gut microbiota of the two samples were. The indicator was positively correlated with alterations in the microbial community of the sample if the point was located in the same direction as the arrow. **L** Spearman’s correlation analysis of liver function indices with the abundance of gut bacteria at the genus level. The darker the color, the higher the correlation was Alanine aminotransferase (ALT), Aspartate aminotransferase(AST), γ-glutamyl transferase (GGT). * p < 0.05, ** p < 0.01, *** p < 0.001

Gut microbiota is involved in APAP-induced liver damage in mice [25], while its dysbiosis is also associated with an increased risk of developing ALF in human [6]. To further determine which bacteria were changed in the gut microbiota of DILI patients with a history of APAP medication, we prospectively collected fecal samples from patients with DILI (DILI, n = 16) and healthy controls (HC, n = 18). The screening flow chart was shown in Supplementary Fig. 1A, and the clinical and demographic characteristics of DILI patients were shown in Supplementary Table 1. The full-length 16S rRNA sequencing was performed to clarify differences in bacterial community. A total of 204 OTUs (20 unique OTUs) and 263 OTUs (79 unique OTUs) were found in DILI and HC, respectively (Fig. 1A). Alpha-diversity indices were decreased in DILI group **(**Fig. 1B-D**)**, which was consistent with previous reports [26, 27]. In terms of beta-diversity, PCoA showed that DILI and HC groups separated significantly (ANOSIM, p = 0.001) (Fig. 1E). Gut microbiome health index (GMHI) is a robust indicator for assessing the health status of gut microbiota, which was higher in HC group (Supplementary Fig. 1B). The GMHI was also strongly and positively correlated with the indices of Sobs, Shannon, and Chao (Supplementary Fig. 1C-E). Meaningful biomarkers were identified using LEfSe (Supplementary Fig. 1F-G). At the phylum level, Proteobacteria increased while Firmicutes and Actinobacteria decreased in DILI group. At the genus level, *Escherichia*, *unclassified_f_Enterobacteriaceae*, and *Roseburia* increased in DILI group, but *unclassified_f_Oscillospiraceae*, *unclassified_o_Eubacteriales*, *Romboutsia*, and *Bifidobacterium* increased in HC group. At the species level, DILI group showed an increase in *Escherichia_fergusonii* and *Clostridium_saudiense*, and HC group exhibited an increase in *Romboutsia_timonensis*, *Mediterraneibacter_faecis*, *Dorea_longicatena* and so on. Bugbase phenotypic prediction was used to further measure the functional composition. Compared to HC group, Gram-negative bacteria increased but Gram-positive and anaerobic bacteria decreased in DILI group (Fig. 1F-G). The top 20 genera contributing strongly to the phenotypes were showed in Fig. 1H-I. Of note, *Bifidobacterium*, often considered as a beneficial genus, decreased significantly in DILI group (Fig. 1J).

The association between clinical indices of liver function (ALT, AST, and GGT, with higher level indicating greater severity of hepatic injury) and gut bacteria was then studied. RDA showed that ALT, AST, and GGT were positively associated with gut bacteria genera from DILI patients and negatively related with HC subjects (Fig. 1K). Spearman correlation analysis revealed that *Escherichia* were positively related with ALT, AST, and GGT, while *Bifidobacterium* was negatively associated with these indicators (Fig. 1L). These results indicated that Gram-negative bacteria, like *Escherichia*, may exacerbate hepatic injury and that inhibiting their growth can help in disease recovery. Previous studies suggested that several probiotics helped inhibit the adherence and pathogenicity of *Escherichia* [28–31]. Thus, we tried to supplement Gram-positive probiotics to clarify whether they were protective in DILI. One clinically commonly used probiotics mixture containing *Bifidobacterium Longum*, *Streptococcus thermophilus*, and *Lactobacillus delbrueckii subspecies bulgaricus* (BSL), may be helpful in reversing drug-induced gut dysbiosis and alleviating hepatic injury. We therewith performed experiments to test this hypothesis and determined underlying mechanisms.

### Supplementation of BSL prevented APAP-induced liver injury

**Fig. 2:**
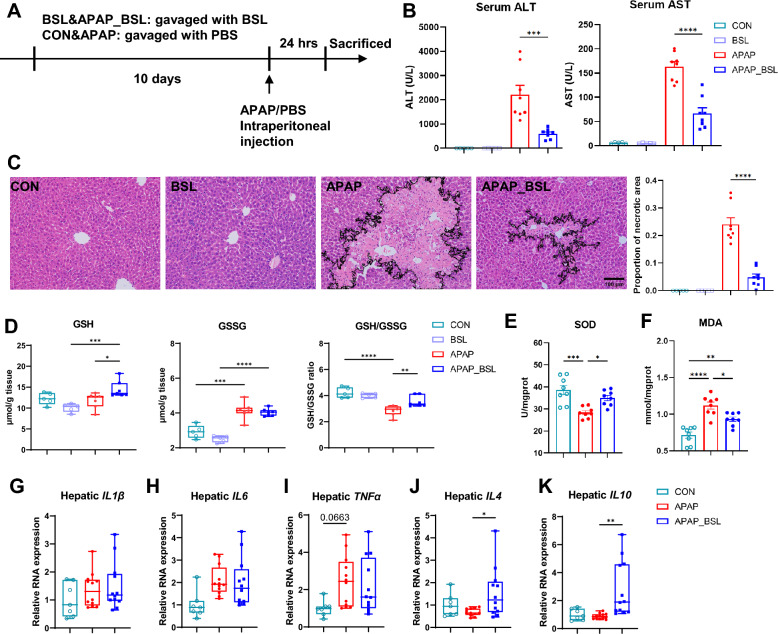
Supplementation of BSL prevented APAP-induced liver injury. **A** Experimental Scheme. **B** Serum ALT and AST decreased significantly in APAP_BSL group compared to APAP group (using Student’s t test). n = 5–8. **C** Representative pictures of H&E staining and quantification of necrotic areas in the liver (scale bar = 100 μm, using Student’s t test). n = 5–8. **D** The levels of hepatic GSH, GSSG, and the GSH/GSSG ratio. n = 5–7. **E** The levels of hepatic SOD in different groups. n = 8. **F** The levels of hepatic MDA in different groups. n = 8. **G**–**K** Relative expression of pro- or anti-inflammatory factors in liver tissues. n = 7–12. One-way ANOVA was used to compare multiple groups if not otherwise specified. * p < 0.05, = ** p < 0.01, *** p < 0.001, **** p < 0.0001

The stable AILI model was conducted based on previous reports [25]. To identify the effect of BSL on AILI, mice were orally pretreated with BSL for 10 days (Fig. 2A). Their body weight did not change during the period of intervention (Supplementary Fig. 2A). Compared to APAP group, liver injury presented as the levels of serum ALT and AST were much lower in APAP_BSL group (Fig. 2B). The area of hepatic necrosis shown in H&E staining in APAP_BSL group decreased markedly (Fig. 2C). The total GSH and GSSG were tested to evaluate hepatic oxidative stress. Compared to Con group, the level of GSH seemed to increase in APAP_BSL group but not in APAP group, and the level of GSSG was also elevated in both APAP and APAP_BSL group. Besides, the ratio of GSH to GSSG was increased significantly in APAP_BSL when compared to APAP group, indicating a decrease of oxidative damage due to BSL administration (Fig. 2D). Moreover, the level of MDA and SOD decreased in APAP group but increased in APAP_BSL group (Fig. 2E, F). BSL supplementation had no additional impacts on the liver, which did not change the levels of hepatic enzymes, histology, and oxidative stress indicators in BSL group compared to those in CON group (Fig. 2B–D). Next, we explored whether protective effects persisted with supplementation of a single probiotic strain, but the results were not favorable. Despite a clear downtrend in ALT, there was almost no change in AST (Supplementary Fig. 2B-C). One single probiotic would lead to limited improvements in AILI, therefore, the subsequent experiments all used BSL to explore AILI alleviation mechanism.

Prior studies demonstrated that inflammatory factors played important roles in AILI and probiotics supplementation usually inhibited their expression [32]. Our results showed that the mRNA levels of pro-inflammatory factors (*IL-1β*, *IL-6,* and *TNF-α*) were unchanged between APAP_BSL and APAP group, however, anti-inflammatory factors (*IL-4* and *IL-10*) increased markedly in APAP_BSL group (Fig. 2G–K), suggesting that BSL might facilitate resistance to inflammatory injury.

Based on previous reports, APAP-induced cell death relies on the level of N-acetyl-p-benzoquinone imine (NAPQI) and its reaction with GSH or protein sulfhydryl groups [33]. GSH is an important antioxidant which can detoxify NAPQI, and the toxic cascades of NAPQI will be initiated owing to the depletion of GSH. Hence, we detected hepatic GSH at the first 30 min after APAP, and found that their levels were very low and no difference was observed between APAP and APAP_BSL group (Supplementary Fig. 2D). The expression of CYP2E1 and CYP1A2, which played key roles in the conversion of APAP to NAPQI [34, 35], showed no significant difference between APAP and APAP_BSL group (Supplementary Fig. 2E), either. The above results indicated that BSL-pretreatment did not increase the levels of detoxicant GSH and metabolic enzymes, thus its protection effect did not function by interfering with APAP early metabolism.

### BSL suppressed the hepatic damage pathway, especially Hippo signaling pathway

**Fig. 3:**
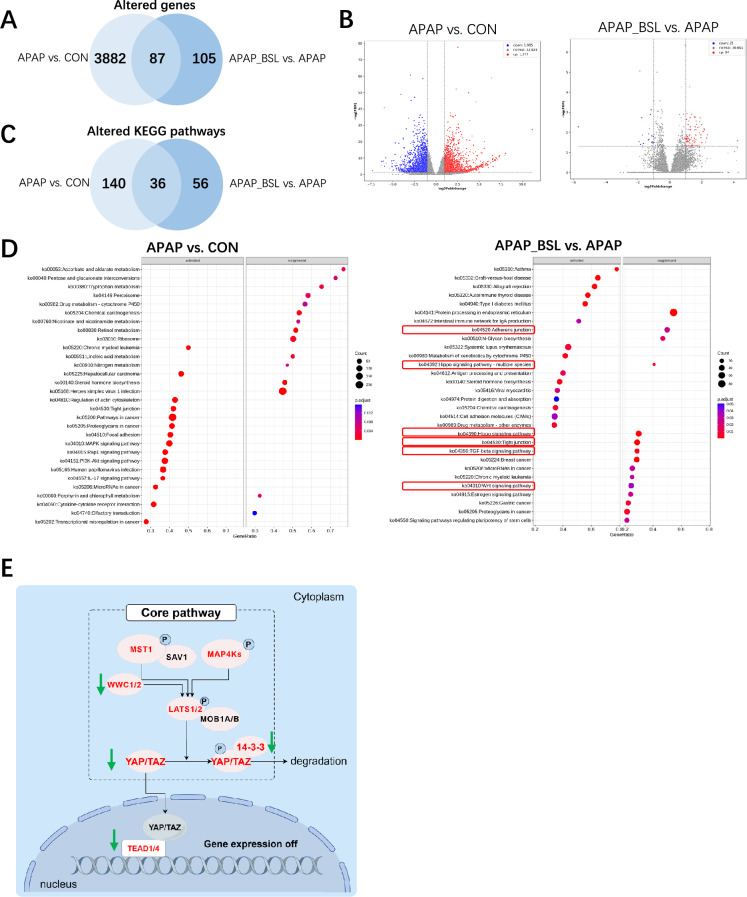
BSL suppressed the hepatic damage pathway, especially Hippo signaling pathway. **A** Venn diagram of significantly altered genes by APAP or by BSL. **B** Volcanic maps of differential genes (padj < 0.05, fold change > 2) between APAP group and CON group, and between APAP_BSL group and APAP group. **C** Venn diagram of significantly enriched KEGG pathways by APAP or by BSL. **D** Top 30 differential KEGG pathways (padj < 0.05) between APAP group and CON group, and between APAP_BSL group and APAP group based on GSEA analysis. Red boxes circled pathways mentioned in paper that were highly expressed in the APAP group. **E** The Hippo signaling pathway. When the pathway was activated, MAP4Ks, MST1/2 and its scaffolding protein SAV1 phosphorylated LATS1/2 and its scaffold MOB1A/B. Activated LATS1/2 phosphorylated YAP and TAZ, preventing their translocation into the nucleus to interact with TEADs to regulate downstream transcription. 14–3-3 protein encoded by *Ywha* bound phosphorylated proteins to keep them in the cytoplasm. Compared to CON group, the genes up-regulated in the APAP group were highlighted in red. Compared to APAP group, genes in the APAP_BSL group that exhibited decreased transcription levels were indicated by green downward arrows. * p < 0.05, ** p < 0.01, *** p < 0.001

To better understand the hepatoprotective role of BSL, RNA-sequencing was applied to liver samples. A total of 3882 and 105 genes differed significantly in CON vs. APAP and in APAP vs. APAP_BSL (padj < 0.05, fold change > 2), respectively (Fig. 3A, B). Among the 87 overlapped genes, 73 genes were downregulated in APAP but upregulated in CON and APAP_BSL groups, and 14 genes were upregulated in APAP but downregulated in other two groups. Gene Set Enrichment Analysis identified 140 and 56 significantly enriched KEGG pathways in CON vs. APAP and in APAP vs. APAP_BSL (p < 0.05 and a false discovery rate q < 0.25 were considered statistically significant), respectively (Fig. 3C, D). Among the overlapped pathways (Supplementary Table 2), twenty-seven pathways, such as Hippo signaling pathway, TGF-β signaling pathway, Wnt signaling pathway, tight and adherens junction, were up-regulated in APAP but down-regulated in CON and APAP_BSL. Seven pathways, like steroid hormone biosynthesis, metabolism of xenobiotics by cytochrome P450, and drug metabolism-cytochrome P450, were down-regulated in APAP group but up-regulated in other two groups.

Notably, the Hippo pathway plays a crucial role in regulating cell growth and development and in mediating tissue regeneration and repair, dysregulation of which can cause liver diseases [36]. Significantly altered genes involved in this signaling pathway were mapped based on their relative expression (Fig. 3E**, **SupplementaryFig. 3A-N). Previous studies confirmed that APAP induced nuclear enrichment of Yes-associated protein (YAP) in hepatocytes [37] and hepatocyte-specific deletion of YAP led to faster recovery from AILI [38]. In this study, the transcription levels of genes associated with Hippo signaling, such as *Yap1* and *Wwc1*, were higher in APAP group than in CON group, while BSL-pretreatment partially reversed the elevated expression owing to APAP. Given such evidence, it was reasonable to speculate that BSL might inhibit the Hippo signaling pathway, especially the expression of YAP. We also focused on the genes (*Nrf2*, *HMOX1*, and *NQO1*) of Nrf2 signaling pathway, which generally act as antioxidants and play protective roles in AILI [39]. No significant difference was observed between APAP and APAP_BSL groups, suggesting their protective effects were limited in our study (Supplementary Fig. 3O-Q). Taken together, BSL may exert protective actions by reversing APAP-induced abnormal damage pathways to near-normal levels.

### BSL ameliorated APAP-induced cecal bacterial dysbiosis

**Fig. 4:**
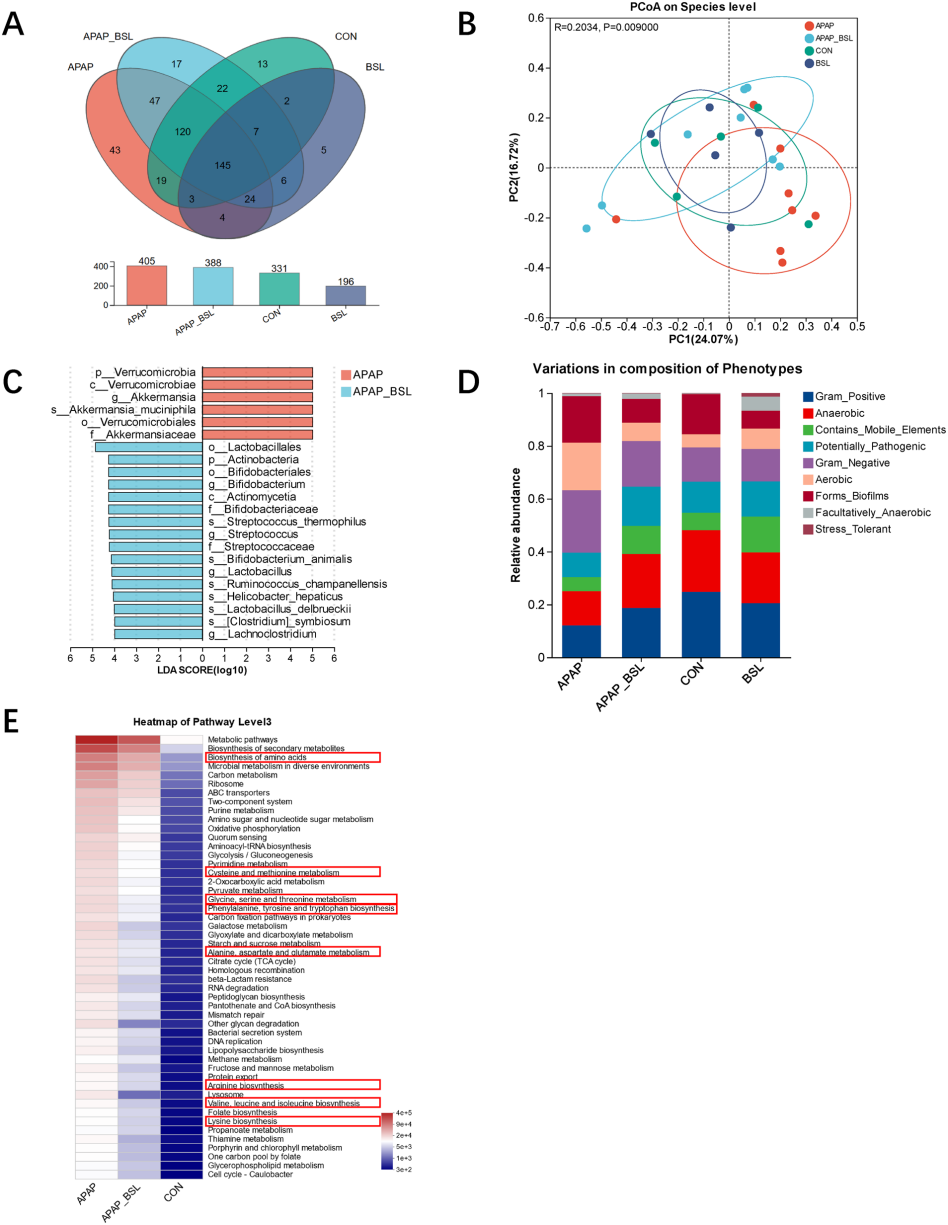
BSL ameliorated APAP-induced cecal bacterial dysbiosis. **A** Venn diagram of APAP, APAP_BSL, CON, and BSL group at the OTU level based on the full-length 16S rRNA sequencing. n = 5–8. **B** PcoA diagram of gut bacteria at the species level (using bray–curtis distance algorithms, anosim analysis compared the difference). **C** Histogram of LDA analysis for APAP group and APAP_BSL group. Microbes with LDA scores (log10) greater than 3 were considered differential microbiota. **D** The phenotypic prediction of four groups based on Bugbase. **E** The heatmap of predicted function of differential gut bacteria based on Picrust2. The metabolic pathways of amino acids were circled by red boxes

To determine the impact of BSL on gut microbiota, full-length 16S rRNA and ITS sequencing of colonic content were performed, respectively. For bacterial community, Venn analysis showed that a total of 331 OTUs with 13 unique OTUs, 196 OTUs with 5 unique OTUs, 405 OTUs with 43 unique OTUs, and 388 OTUs with 17 unique OTUs were identified in the CON, BSL, APAP and APAP_BSL group, respectively (Fig. 4A). BSL-pretreatment significantly changed beta-diversity of gut bacteria rather than alpha-diversity. The observed species, Shannon, and Chao indices at the species level were not markedly different between APAP group and APAP_BSL group (Supplementary Fig. 4A-C). PCoA showed that BSL made the gut bacterial community of APAP_BSL group more similar to that of CON group at the species level (ANOSIM, p = 0.009) (Fig. 4B). The difference of beta-diversity between APAP group and APAP_BSL group was significant based on ANOSIM (p = 0.04). LEfSe was performed to find differential microbiota between APAP group and APAP_BSL group. At the phylum level, the abundance of *Verrucomicrobia* enriched but *Actinobacteria* decreased in APAP group. At the genus level, there was an increase of *Akkermansia* and a decrease of *Bifidobacterium*, *Streptococcus*, *Lactobacillus*, and *Lachnoclostridium* in the APAP group. At the species level, the number of *Streptococcus thermophilus*, *Bifidobacterium animalis*, *Lactobacillus delbrueckii*, and *[Clostridium] symbiosum* was increased in APAP_BSL compared to APAP group, while that of *Akkermansia muciniphila* was reduced (Fig. 4C).

Similarly, the fungal community was analyzed. Venn analysis showed that a total of 32 OTUs with 2 unique OTUs, 43 OTUs with 12 unique OTUs, 61 OTUs with 11 unique OTUs, and 57 OTUs with 13 unique OTUs were identified in the CON, BSL, APAP and APAP_BSL group, respectively (Supplementary Fig. 4D). The observed species, Shannon, and Chao indices at the species level were not markedly different between APAP group and APAP_BSL group (Supplementary Fig. 4E-G). Of note, differences in alpha-diversity existed mainly between CON and BSL group, which implied that BSL might promote the growth of certain fungi. In terms of beta-diversity, gut fungi in APAP and APAP_BSL group cannot be distinguished by PCoA at the species level (ANOSIM, p = 0.561) (Supplementary Fig. 4H), indicating that the protective effect of BSL was unlikely to be functioned through gut mycobiota.

Based on Bugbase phenotypic predictions, anaerobic and Gram-positive bacteria in APAP group exhibited a decreasing trend, while BSL-pretreatment can reverse this situation, which was consistent with findings observed in our DILI patients (Fig. 1F, 4D). PICRUSt2 analysis was used to deepen understanding of the potential function of differential gut bacteria. Supplementation of BSL significantly changed metabolism related pathways, especially amino acids metabolism (Fig. 4E). For instance, the biosynthesis of amino acids, such as valine, leucine, isoleucine, and lysine, was decreased in APAP_BSL group compared to APAP group. Taken together, APAP mainly affected beta-diversity of intestinal bacteria, while BSL attenuated APAP-induced gut dysbiosis and played a protective role probably through altering gut-derived amino acids. We then tested the cecum metabolome to further verify the predicted results.

### BSL altered metabolite profiles and inhibited oligopeptides containing branched-chain amino acids

**Fig. 5:**
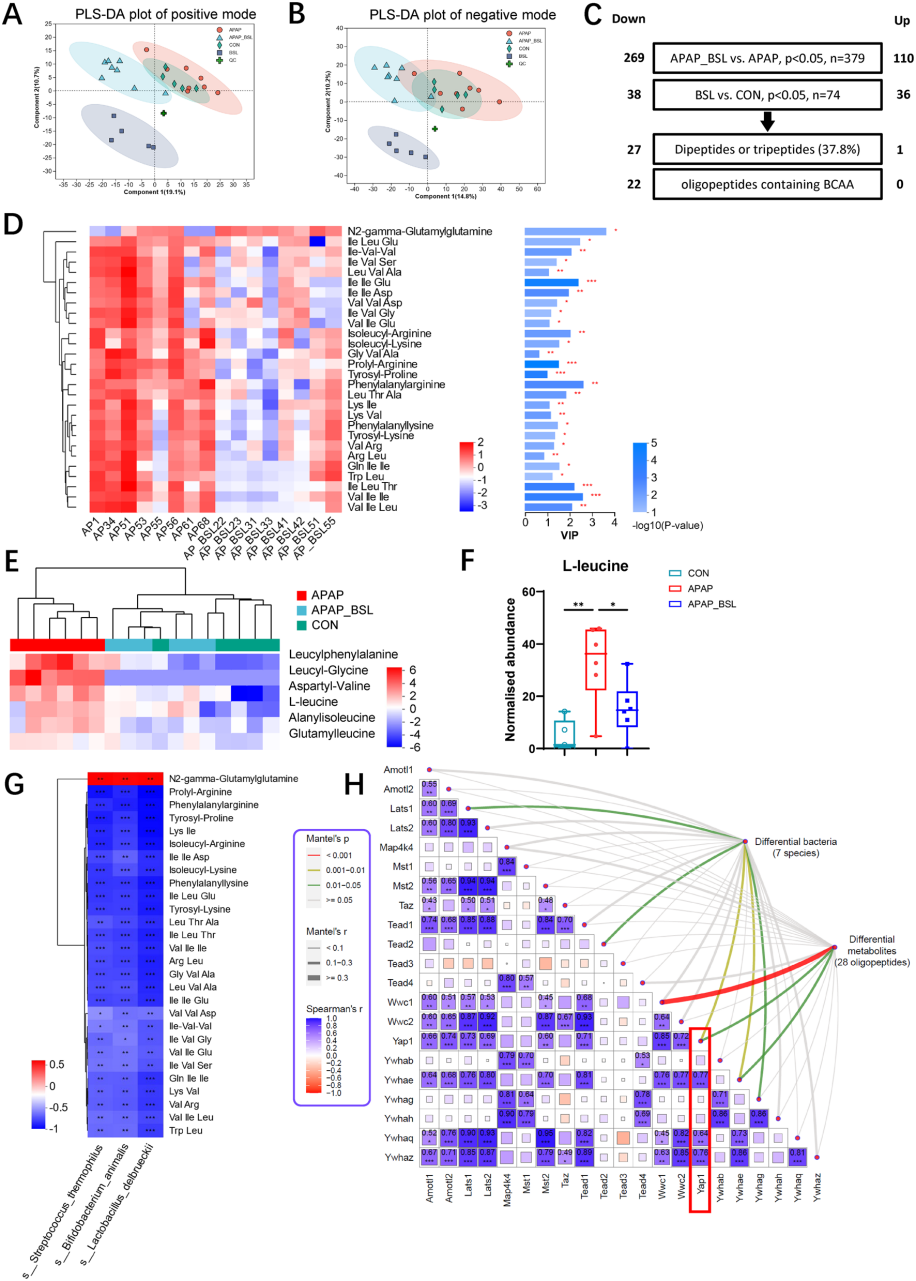
BSL altered metabolite profiles and inhibited oligopeptides containing branched-chain amino acids. **A**-**B** PLS-DA plot of four groups metabolites in positive and negative mode, respectively. QC, quality control. n = 5–8. **C** The strategies of screening differential metabolites (p < 0.05, using Student’s t test). **D** Heatmap of intestinal differential metabolites and their VIP values. n = 8. **E** Heatmap of hepatic differential metabolites. n = 5–6. **F** The hepatic level of L-leucine based on metabolome. n = 5–6. **G** Spearman’s correlation heatmap between bacteria and oligopeptides with significant differences. **H** Pairwise comparisons of key genes in the Hippo signaling pathway were shown, with a color gradient denoting Spearman’s correlation coefficient. Differential bacteria and metabolites were related to each genes using Mantel-test analysis. Edge width corresponded to the Mantel’s r statistic for the corresponding distance correlations, and edge color represented the statistical significance. * p < 0.05, ** p < 0.01, *** p < 0.001

Untargeted metabolomic analysis was performed on the cecal contents based on LC–MS/MS. A total of 2072 metabolites were detected (862 positive ions and 1210 negative ions). According to PLS-DA, there was an obvious separation in both positive and negative modes between APAP group and APAP_BSL group (Fig. 5A, B). Validation plots showed no over-fitting in the process of model generation (Supplementary Fig. 5A-B). So, it was reasonable to deduce that: i) the characteristics of cecal metabolites were changed obviously after probiotics supplementation; ii) certain metabolites in BSL-pretreated mice may improve AILI.

To find cecal metabolites potentially associated with the differential APAP hepatoxicity, data were filtered through following selection criteria (Fig. 5C). First, metabolites with statistical significance (p < 0.05) between APAP group and APAP_BSL group were selected. Second, differential metabolites (p < 0.05) between CON group and BSL group were also selected, while those with inconsistent changes between the two comparison groups were excluded. As a result, 74 metabolites shared same variation tendency after BSL supplementation, of which 38 were down-regulated and 36 were up-regulated, which are shown in Supplementary Table 3. Of note, over one-third of compounds (twenty-eight) are classified as dipeptides or tripeptides, with 22 of them containing BCAAs (Fig. 5D). All oligopeptides above were reduced in APAP_BSL group except for N2-gamma-Glutamylglutamine (N2-γ-GluGln). To further explore BCAA alterations in the liver, the hepatic metabolome was then conducted. Significant separation was observed between APAP group and APAP_BSL group in both positive- and negative- ion modes (Supplementary Fig. 5C-D), indicating that BSL treatment also altered the hepatic metabolites. Some oligopeptides containing BCAAs did display a decreasing trend in APAP_BSL group compared to APAP group (Fig. 5E). Notably, the normalized abundance of L-leucine, in particular, was highest in APAP group, but isoleucine and valine were not detected (Fig. 5F). Interestingly, the BCAAs biosynthesis was decreased in APAP_BSL group according to the PICRUSt2 prediction (Fig. 4E), and the short chain peptides containing BCAAs also reduced in cecum and liver of APAP_BSL group when compared to APAP group, suggesting the consistency of microbiome and metabolome.

### High correlativity among multi-omics results: BSL-oligopeptides-Hippo pathways

Given that BSL altered the structure of intestinal bacteria and metabolite profiles, correlation analyses were subsequently performed. Spearman’s correlation analysis showed that differential bacterial strains were closely related to differential metabolites (**Supplementary Fig. 6A**). In particular, *Bifidobacterium animalis*, *Streptococcus thermophilus*, and *Lactobacillus delbrueckii* were significantly negatively associated with 22 oligopeptides containing BCAAs and positively correlated with N2-γ-GluGln (Fig. 5G). Notably, glutamine was required for the production of N2-γ-GluGln, so the elevation of this metabolite indicated the increased abundance of glutamine in APAP_BSL group. Glutamine can preserve hepatic GSH and reduce tissue injury caused by APAP [40, 41]. Hence, it was reasonable to assume that BSL might increase protective metabolites on the one hand and inhibit BCAA-containing short peptides on the other, both of which improved AILI ultimately. We next focused our research on metabolites that may have damaging effects.

Small metabolites originating from intestine can enter the blood circulation and reach the liver through the portal vein, potentially causing alterations in the expression of hepatic genes. Hence, we further linked the gut results to the expression of Hippo signaling genes using Spearman’s correlation analyses and Mantel-test analysis (Fig. 5H). The results showed that *Yap1* and *Ywhae*, key genes in activated Hippo pathway, were correlated with other two matrices (for *Yap1*, differential bacteria profiles: Mantel’s r = 0.240 and differential metabolites profiles: Mantel’s r = 0.253; for *Ywhae*, differential bacteria profiles: Mantel’s r = 0.285, and differential metabolites profiles: Mantel’s r = 0.205; all p < 0.05, their roles in Hippo were illustrated in Fig. 3E), and the two genes were also highly positively correlated (Spearman’s r = 0.85, p < 0.001), which was consistent with our hypothesis that differences in our transcriptome results can be partially explained by the gut microbiome and metabolome. Overall, the levels of short chain peptides containing BCAAs in the cecum increased markedly after APAP administration. They will reach liver and be degraded into valine, leucine, isoleucine, and other amino acids, and these excess compounds were likely to activate Hippo signaling and synergistically exacerbate liver injury. Thus, we next explored the effect of excess BCAAs on liver cells in both normal and injured states.

### Supplementation of leucine aggravated AILI

**Fig. 6:**
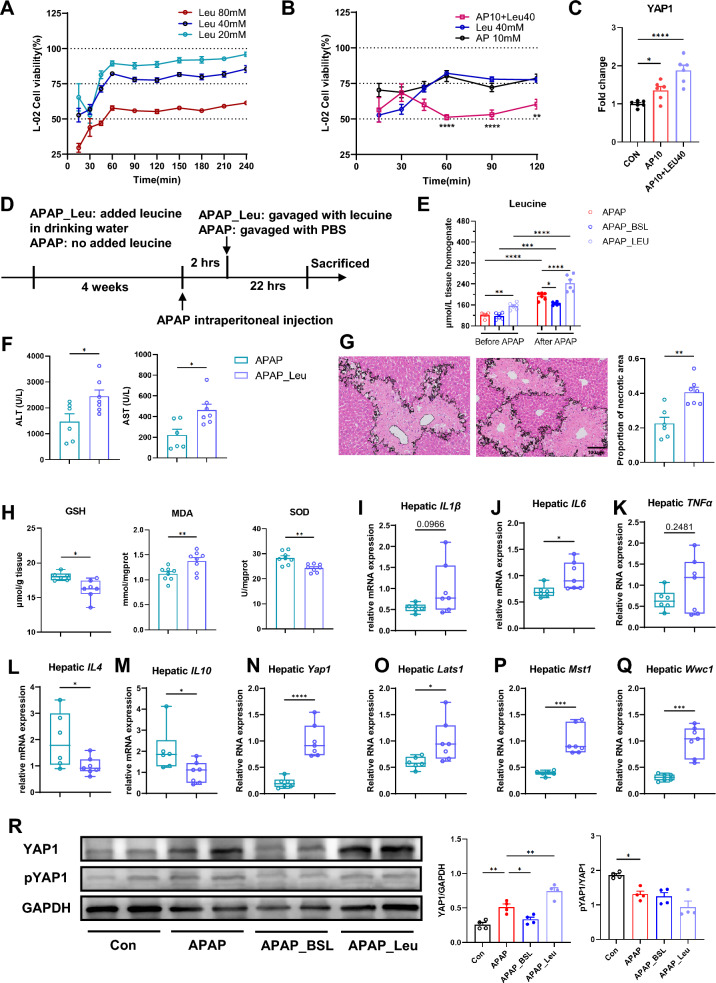
Supplementation of leucine aggravated AILI. **A** Effects of different concentrations of leucine on cell viability (using one-way ANOVA). The viability in cells added only with CCK8 was 100% (not shown in the figure). n = 6. **B** Leucine exacerbated the inhibition of hepatocyte viability by APAP (using two-way ANOVA). The viability in cells added only with CCK8 was 100% (not shown in the figure). * indicated a significant difference between AP10 + Leu40 and AP10. n = 6. **C** The mRNA expression of YAP1 in L-02 cells. n = 6. **D** Experimental Scheme for supplementation of leucine. **E** The hepatic level of Leucine in different groups. n = 6. **F** Serum ALT and AST increased significantly in APAP_Leu group when compared to APAP group. n = 6–7. **G** Representative pictures of H&E staining and quantification of necrotic areas in the liver (scale bar = 100 μm). n = 6–7. **H** The levels of hepatic GSH, MDA, and SOD in two groups. n = 6–7. **I**–**M** Relative expression of pro- and anti-inflammatory factors in liver tissues. n = 6–7. **N**–**Q** Relative expression of genes in Hippo signaling pathways in liver tissues. n = 6–7. **R** The protein expression of YAP1 in different groups. Student’s t test was used to compare the two groups if not otherwise specified. * p < 0.05, ** p < 0.01, *** p < 0.001, **** p < 0.0001

The L-02 cell lines were used to assess the viability of hepatocytes to different concentrations of APAP and BCAAs. Cells were starved for 12 h before intervention. 15mM was the half maximal inhibitory concentration of APAP (Supplementary Fig. 7A), which is consistent with previous reports [10]. Hepatocyte viability was mildly reduced by isoleucine or valine at concentrations of 40mM and 80mM, respectively (Supplementary Fig. 7B). However, high (40 or 80mM) but not low concentrations (20 mM) of leucine significantly inhibited the proliferation of hepatocytes (Fig. 6A). Then, we incubated hepatocytes with APAP (10mM) and three kinds of BCAAs (40mM) for 24h, respectively. The results revealed that simultaneous addition of leucine and APAP showed more pronounced inhibition of hepatocytes proliferation (Fig. 6B). The cell viability dropped from 75% to about 60%. The combination of isoleucine and APAP slightly increased inhibitory effects, whereas the combination of valine and APAP showed no discernible impact (Supplementary Fig. 7C). Moreover, the mRNA level of YAP1 was significantly increased in APAP_Leu group compared to APAP group (Fig. 6C). Thus, leucine was more likely to aggravate the AILI compared to valine and isoleucine by promoting YAP1 expression.

To validate the damaging role of leucine, we added it (1.5% w/v) to drinking water for four weeks before APAP exposure (Fig. 6D). Before APAP administration, the level of Leu in APAP_Leu group was increased significantly. After 24 h of APAP injection, the concentration of Leu increased in all groups. Its level remained highest in APAP_Leu group, followed by APAP group, and lowest in APAP_BSL group (Fig. 6E). The serum ALT and AST were not elevated before administration of APAP (**Supplementary Fig. 7D**), indicating that leucine at this concentration did not cause additional liver damage. 24 h after intraperitoneal injection of APAP, the levels of hepatic enzymes (Fig. 6F) and the necrotic area of liver (Fig. 6G) were elevated significantly in APAP_Leu group, suggesting that leucine exacerbated liver injury during AILI. The level of GSH and SOD decreased significantly in APAP_Leu group, but MDA increased (Fig. 6H). Quantitative real-time PCR showed that the relative mRNA expression of IL-6 increased, while the levels of anti-inflammatory, IL-4 and IL-10, decreased in APAP_Leu group (Fig. 6I–M**)**, suggesting that leucine led to increased oxidative stress and inflammation.

### Leucine upregulated YAP and exacerbated AILI

Previous studies have confirmed that the upregulation of YAP/TAZ-mediated signaling promoted the progression of AILI [42]. To explore whether leucine exacerbated the AILI through the Hippo signaling pathway, the mRNA expressions of *Yap1*, *Lats1*, *Mst1*, and *Wwc1*, were detected in mice model, and they were elevated significantly in APAP_Leu group compared to APAP group (Fig. 6N–Q). Besides, the protein expression of YAP1 was highest in APAP_Leu group, followed by the APAP group, with lower expression in the Con and APAP_BSL groups. There was no significant difference between APAP-treated groups in the levels of phosphorylated YAP1 (Fig. 6R). To further validate the role of YAP in AILI model, we used Verteporfin, a kind of YAP inhibitor, before APAP administration (Supplementary Fig. 7E). The results showed that VP could ameliorate AILI liver injury (Supplementary Fig. 7F-J). All above results indicated that leucine might worsen hepatic injury through activating the YAP1-mediated Hippo signaling pathway.

## Discussion

In this study, we investigated the potential impact of probiotic supplementation on acute liver injury induced by APAP and explored protective mechanisms. Our finding revealed that APAP administration led to alterations in amino acids metabolism and metabolite profiles in the gut and liver. In particular, the levels of short peptides containing BCAAs increased following APAP administration. Moreover, we observed that leucine exacerbated hepatocyte injury through activating the Hippo signaling pathway, especially the overexpression of YAP1, while probiotics composed of Gram-positive bacteria could reverse this process.

The role of the Hippo signaling in AILI has been previously explored. When the Hippo pathway is activated, MAP4Ks, MST1/2 and the scaffolding protein SAV1 phosphorylate LATS1/2 and its scaffold MOB1A/B. Activated LATS1/2 phosphorylates YAP and TAZ, preventing them from translocating to the nucleus to interact with TEADs (Fig. 3E) [43]. Inhibition of the *Mst1*/*2*, the upstream genes of *Yap1*, can promote liver repair [44]. Similarly, hepatic-specific knockout of the *Yap* promotes an accelerated recovery from AILI [38]. Hence, inhibiting the Hippo pathway and the expression of YAP seems to be a promising therapeutic strategy. However, a recent study has suggested that multiple polysaccharides can decrease gut permeability, upregulate YAP expression, and ultimately contribute to the repair of AILI [45]. In our study, we found a correlation among the expression of *Yap1*, the abundance of probiotics and short peptides containing leucine (Fig. 5H). The mice supplemented with leucine exhibited an upregulation in the relative abundance of Hippo-related genes, and their hepatic injury was more pronounced after APAP injection. Nevertheless, further exploration is required to elucidate the mechanisms through which leucine regulates this pathway.

It was reported in 2009 that gut microbiota in humans was associated with the metabolism of APAP [46]. Subsequently, a large number of studies have shown that beneficial bacteria, such as *Bifidobacterium* and *Lactobacillus*, can ameliorate AILI [10, 47]. Notably, most of the supplemented strains belonged to Gram-positive bacteria. Recent research concluded that gut microbiota dysbiosis in AILI mice was dominated by Gram-negative bacteria enrichment [9], which was further supported by our results of sequencing on human and mouse fecal specimens. The degree of hepatic injury was negatively correlated with a range of Gram-positive bacteria as well. One recent paper reported that *A. muciniphila*, a type of Gram-negative bacterium, can also attenuate AILI through activating PI3K/Akt signaling pathway [48]. However, this strain was significantly higher in APAP group compared to APAP_BSL group, but there was no difference between APAP and CON group (Fig. 4C**, Supplementary Fig. 8A**). Therefore, the possible interpretation was that supplemented BSL competed with *A. muciniphila*, resulting in a significant decrease of its relative abundance in APAP_BSL group, but this phenomenon did not mean that *A. muciniphila* was a harmful bacterium. Furthermore, a recent study revealed that β-Galactosidase, produced by* S. thermophilus*, played a vital role in upregulating the abundance of *Bifidobacterium* and *Lactobacillus*, while simultaneously downregulating the expression of proteins in the Hippo signaling pathway and mediating anticancer effects [49]. Additionally, mice supplemented with a probiotic mixture containing *Lactobacilli*, *Bifidobacteria*, and *Streptococcus salivstius* can also attenuate nonalcoholic steatohepatitis via modulating hepatic genes associated with the Hippo pathway [50]. These results further supported the correlation between microbiome and transcriptomic results in our study (Fig. 5H). In conclusion, probiotics, especially a combination of Gram-positive bacteria, may be a promising strategy for the prevention and treatment of AILI.

Prior studies have reported that several bacteria influencing the metabolism of BCAAs were involved in the development of various metabolic diseases. For example, the genus *Bacteroides*, including *Bacteroides dorei* and *Bacteroides vulgatus*, could promote BCAA catabolism and attenuate mice obesity [51]. *Bacteroides stercoris* promoted non-alcoholic fatty liver disease partially through increasing levels of valine and isoleucine [52]. Administration of *Parabacteroides merdae* enhanced the degradation of intestinal BCAAs, thereby relieving obesity-associated atherosclerosis [53]. However, no significant difference in the above bacteria was observed between APAP group and APAP_BSL group in this study (Supplementary Fig. 8B-C**)**. The relationship between BSL and the metabolism of BCAAs has also been rarely reported. An earlier study showed that the ilvC gene-mediated BCAA synthesis pathway contributed to the optimal growth of *S. thermophilus* in milk [54], but whether this strain could utilize BCAAs is not yet clear. A study in an Indian population suggested that *Bifidobacterium* was associated with BCAA transport and leucine degradation, resulting in higher fecal concentrations of BCAAs but lower serous BCAAs levels [55]. We also observed an elevation in N2-γ-GluGln in APAP mice supplemented with BSL. γ-GluGln belongs to the γ-glutamyl dipeptide, which is usually derived from extracellular GSH degradation. γ-glutamyl transpeptidase can catalyze the synthesis of γ-glutamyl moiety released from GSH with other high concentrations of amino acids [56]. Although the biological role of γ-GluGln is not clear, its formation highly depends on GSH metabolism, indirectly indicating differences in GSH levels between APAP group and APAP_BSL group. Furthermore, BCAAs can be used as a nitrogen source for the synthesis of glutamic acid and glutamine in the muscle and brain [57], which seems to explain why BCAAs-containing peptides were down, while glutamine-containing peptides were up. Taken together, BSL helped to change the metabolism of BCAAs in the intestine, and might contribute to converting them to glutamine, but which bacteria had these functions deserved further study.

The liver is an important organ for amino acid metabolism. It can metabolize all amino acids except for BCAAs because the branched chain amino transferases (BCATs) are barely expressed in hepatocytes [58]. Generally, BCAAs are clinically considered as a kind of protective agent [59]. A meta-analysis of patients with liver cirrhosis found that BCAAs reduced cirrhotic complications [60]. However, some evidence suggests no benefits of BCAAs in the treatment and prognosis of liver diseases. A recent meta-analysis, which enrolled 54 clinical trials, concluded that supplementation of BCAAs had neither a clear benefit on liver function and prognosis nor led to serious side effects [61]. In preclinical research, BCAAs have been shown to improve hepatic steatosis in mice with nonalcoholic steatohepatitis [62] or in rats with carbon tetrachloride-induced cirrhosis [63], but they can also exacerbate NAFLD and obesity [52, 64]. These seemingly conflicting results suggested that the role of BCAAs in liver disease is complex and deserves further study. In our study, leucine supplementation alone did not exacerbate liver injury in mice (Supplementary Fig. 7D), however, in the case of acute liver injury, excessive leucine aggravated hepatic inflammation and necrosis, suggesting that future BCAA should be supplemented with caution in patients with acute liver injury like DILI.

We acknowledge some limitations within this research. Firstly, most liver injury events occur owing to herbal medicines rather than APAP in China. Therefore, the results of 16S rRNA sequencing do not perfectly represent the altered microbiota of AILI patients. Even so, the microbiota exhibits similar characteristics and functions in both mice and human with acute liver injury, such as a decrease in the abundance of Gram-positive and anaerobic bacteria. Besides, we cannot completely exclude the effect of autoimmune hepatitis (AIH) on the gut microbiota due to the difficulty of differential diagnoses of DILI and drug-induced AIH. Given that this is a single-center clinical trial, it is not easy for us to enroll a sufficient number of patients with AILI. In the future, a multi-center study may help to address this issue. Secondly, our study does not explore the direct effect of certain BCAA-containing oligopeptides on the liver. This is mainly because, on one hand, the oligopeptides are absorbed via the intestinal tract and they would be further broken down into amino acids in the blood for the body to utilize. On the other hand, the number of altered oligopeptides is large, and the use of only one kind of them might have a limited effect in the animal model. So, whether BCAA-containing peptides have potential effects on liver cells remains to be explored. Thirdly, the increase in BCAA-containing oligopeptides in the cecum indicates a decrease in BCAA catabolism, and which bacteria contribute to their degradation, especially leucine metabolism, needs to be further investigated.

## Conclusions

In summary, the gut microbiota of DILI patients was disturbed, with a marked decline in Gram-positive bacteria. Pretreatment of mice with BSL can alleviate AILI, improve gut microbiota dysbiosis, alter cecum metabolite profiles, and suppress the hepatic Hippo signaling pathway. The level of probiotics was significantly negatively associated with the presence of oligopeptides containing BCAAs. Interestingly, valine and isoleucine did little to inhibit the proliferation of hepatocytes except for leucine. In vivo experiments further suggested that an administration of leucine exacerbated the process of AILI through activating the Hippo signaling pathway. In the future, maintaining homeostasis of gut microbiota and amino acid metabolism may be important for improving AILI.

## Supplementary Information

Supplementary material 1.

Supplementary material 2.

Supplementary material 3.

Supplementary material 4.

Supplementary material 5.

Supplementary material 6.

## Data Availability

The transcriptome and microbiome data were deposited in the NCBI SRA (PRJNA1066211, PRJNA1065322). The metabolomic data were deposited in the MetaboLights (MTBLS9447).

## Abbreviations

AIH: Autoimmune hepatitis
AILI: APAP-induced liver injury
ALF: Acute liver failure
ALT: Alanine aminotransferase
APAP: Acetaminophen
AST: Aspartate aminotransferase
BCAAs: Branched-chain amino acids
BCATs: Branched chain amino transferases
CYP1A2: Cytochrome p450 1A2
CYP2E1: Cytochrome p450 2E1
FBS: Fetal bovine serum
FPKM: Fragments Per Kilobase Per Million bases
GMHI: Gut microbiome health index
GSH: Glutathione
GSSG: Oxidative glutathione
HC: Healthy control
LEfSe: Linear discriminant analysis effect size
N2-γ-GluGln: N2-gamma-Glutamylglutamine
NAPQI: N-acetyl-p-benzoquinone imine
PBS: Phosphate-buffered saline
PCoA: Principal coordinate analysis
PICRUSt2: Phylogenetic Investigation of Communities by Reconstruction of Unobserved States
PLS-DA: Partial Least Squares Discriminant Analysis
QC: Quality control
RDA: Redundancy analysis
SEM: Standard error of the mean
YAP: Yes-associated protein
