# Supplementary material for: Gram-positive probiotics improves acetaminophen-induced hepatotoxicity by inhibiting leucine and Hippo-YAP pathway
Source: Cell Biosci. 2025 Mar 7;15:32. doi: 10.1186/s13578-025-01370-5 (PMC11887100; doi:10.1186/s13578-025-01370-5)

Supplementary Figure 1

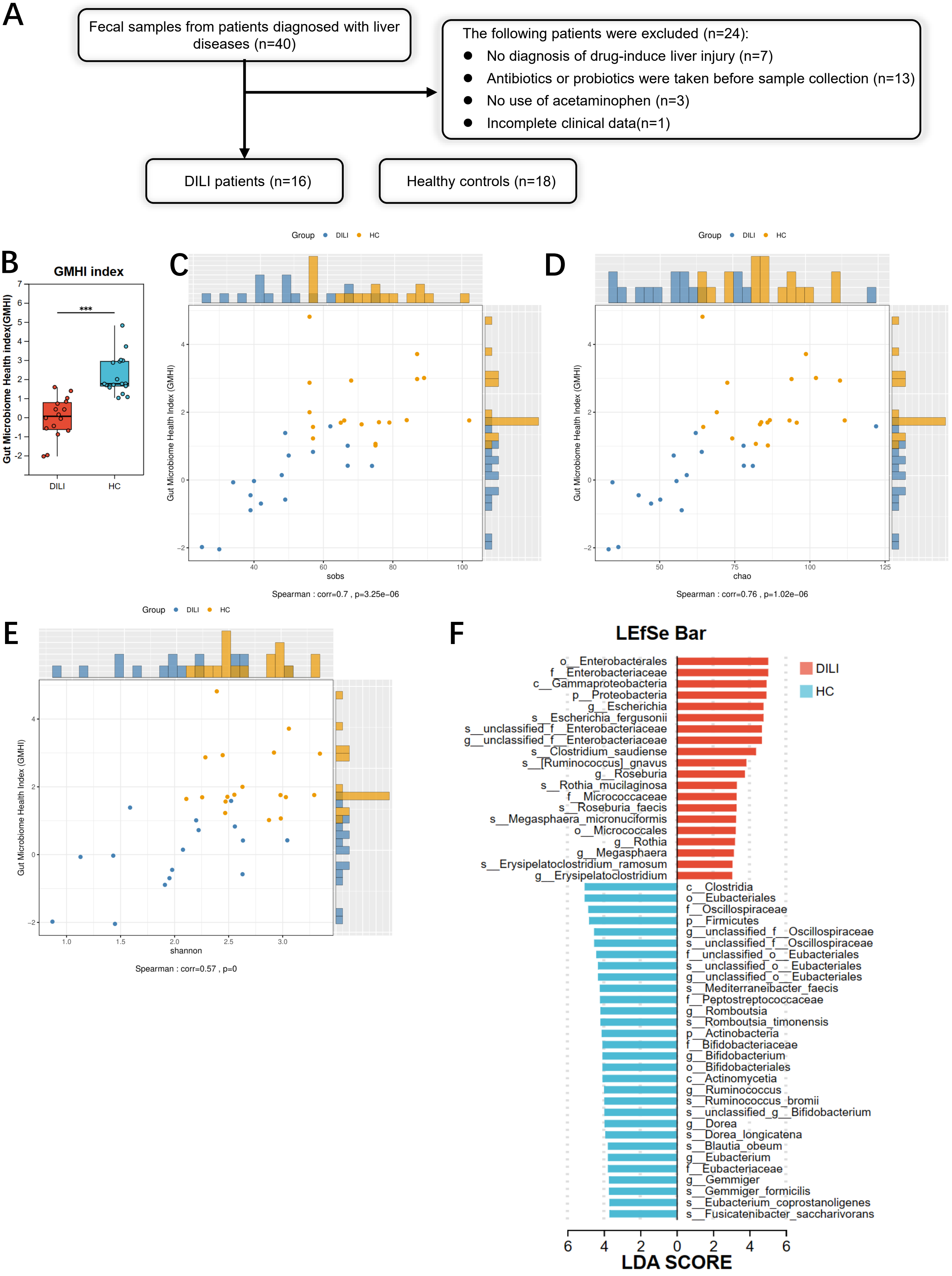

Supplementary Figure 1

G

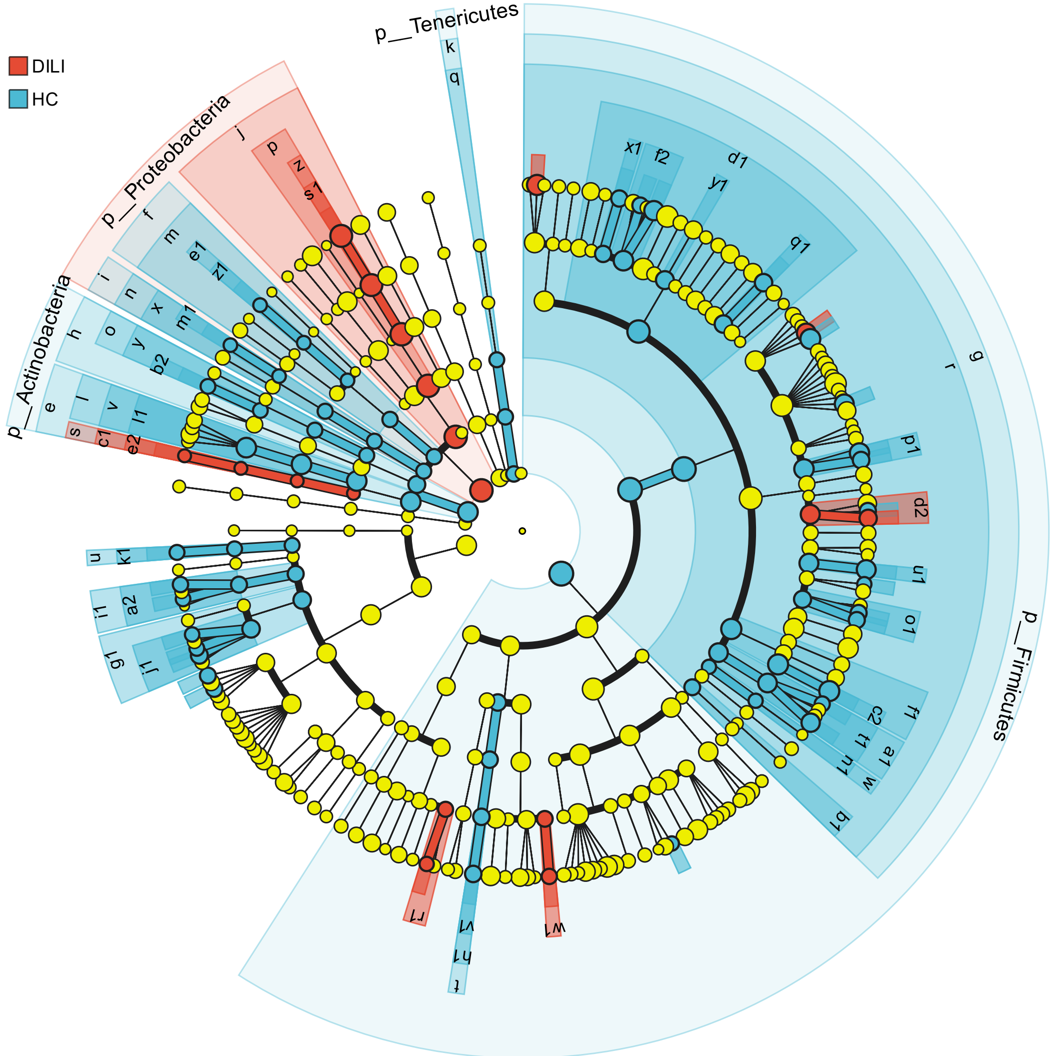

a : p__Actinobacteria
b : p__Firmicutes
c : p__Proteobacteria
d : p__Tenericutes
e : c__Actinomycetia
f : c__Betaproteobacteria
g : c__Clostridia
h : c__Coriobacteriia
i : c__Deltaproteobacteria
j : c__Gammaproteobacteria
k : c__Mollicutes
l : o__Bifidobacteriales
m : o__Burkholderiales
n : o__Desulfovibrionales
o : o__Eggerthellales
p : o__Enterobacterales
q : o__Entomoplasmatales
r : o__Eubacteriales
s : o__Micrococcales
t : o__Selenomonadales
u : f__Barnesiellaceae
v : f__Bifidobacteriaceae
w : f__Christensenellaceae
x : f__Desulfovibrionaceae
y : f__Eggerthellaceae
z : f__Enterobacteriaceae
a1 : f__Eubacteriaceae
b1 : f__Eubacteriales_Family_XIII__Incertae_Sedis
c1 : f__Micrococcaceae
d1 : f__Oscillospiraceae
e1 : f__Oxalobacteraceae
f1 : f__Peptostreptococcaceae
g1 : f__Rikenellaceae
h1 : f__Selenomonadaceae
i1 : f__Tannerellaceae
j1 : g__Alistipes
k1 : g__Barnesiella
l1 : g__Bifidobacterium
m1 : g__Bilophila
n1 : g__Christensenella
o1 : g__Coprococcus
p1 : g__Dorea
q1 : g__Dysosmobacter
r1 : g__Erysipelatoclostridium
s1 : g__Escherichia
t1 : g__Eubacterium
u1 : g__Fusicatenibacter
v1 : g__Megamonas
w 1 : g__Megasphaera
x1 : g__Neglectibacter
y1 : g__Oscillibacter
z1 : g__Oxalobacter
a2 : g__Parabacteroides
b2 : g__Raoultibacter
c2 : g__Romboutsia
d2 : g__Roseburia
e2 : g__Rothia
f2 : g__Ruminococcus

Supplementary Figure 2

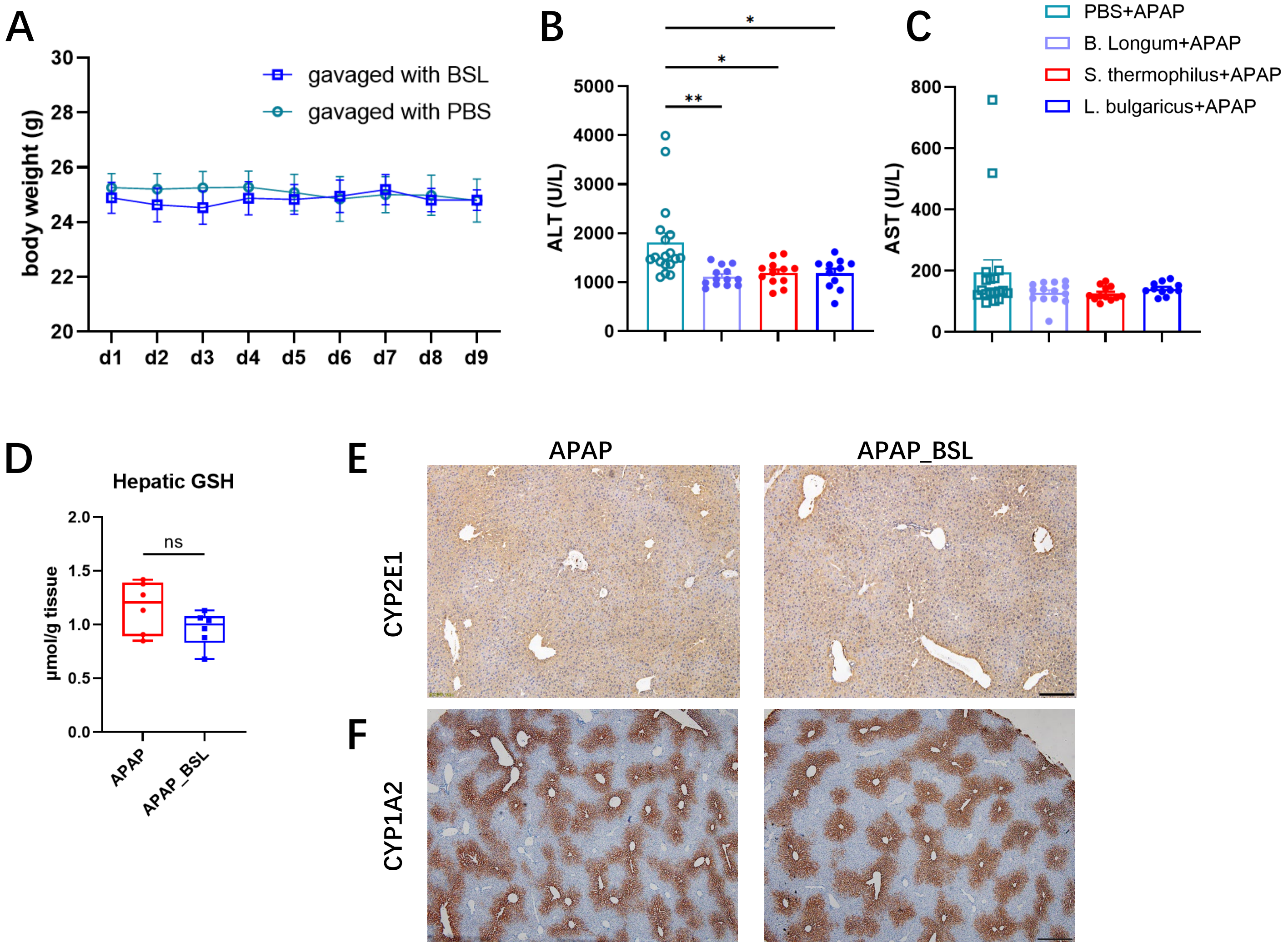

Supplementary Figure 3

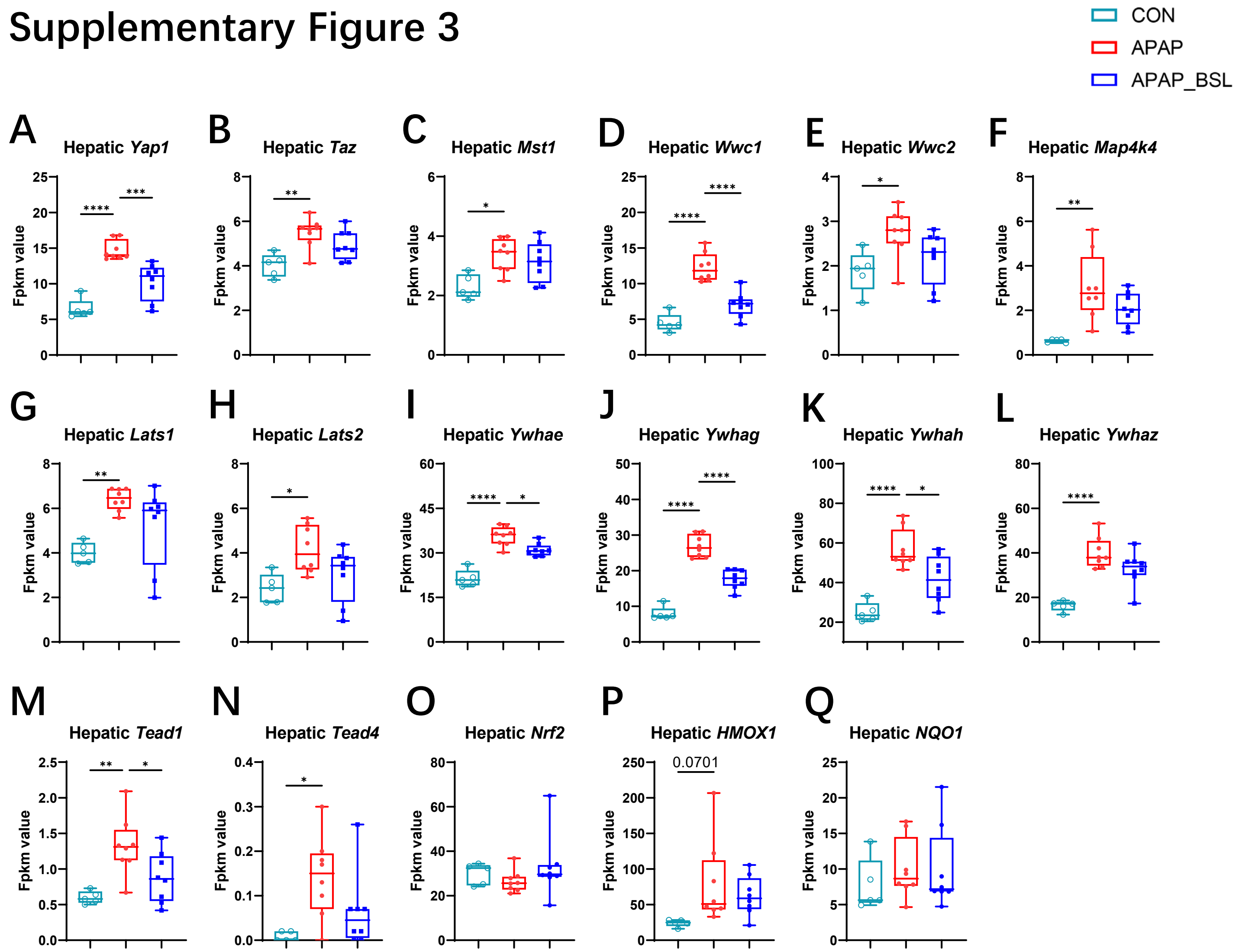

Supplementary Figure 4

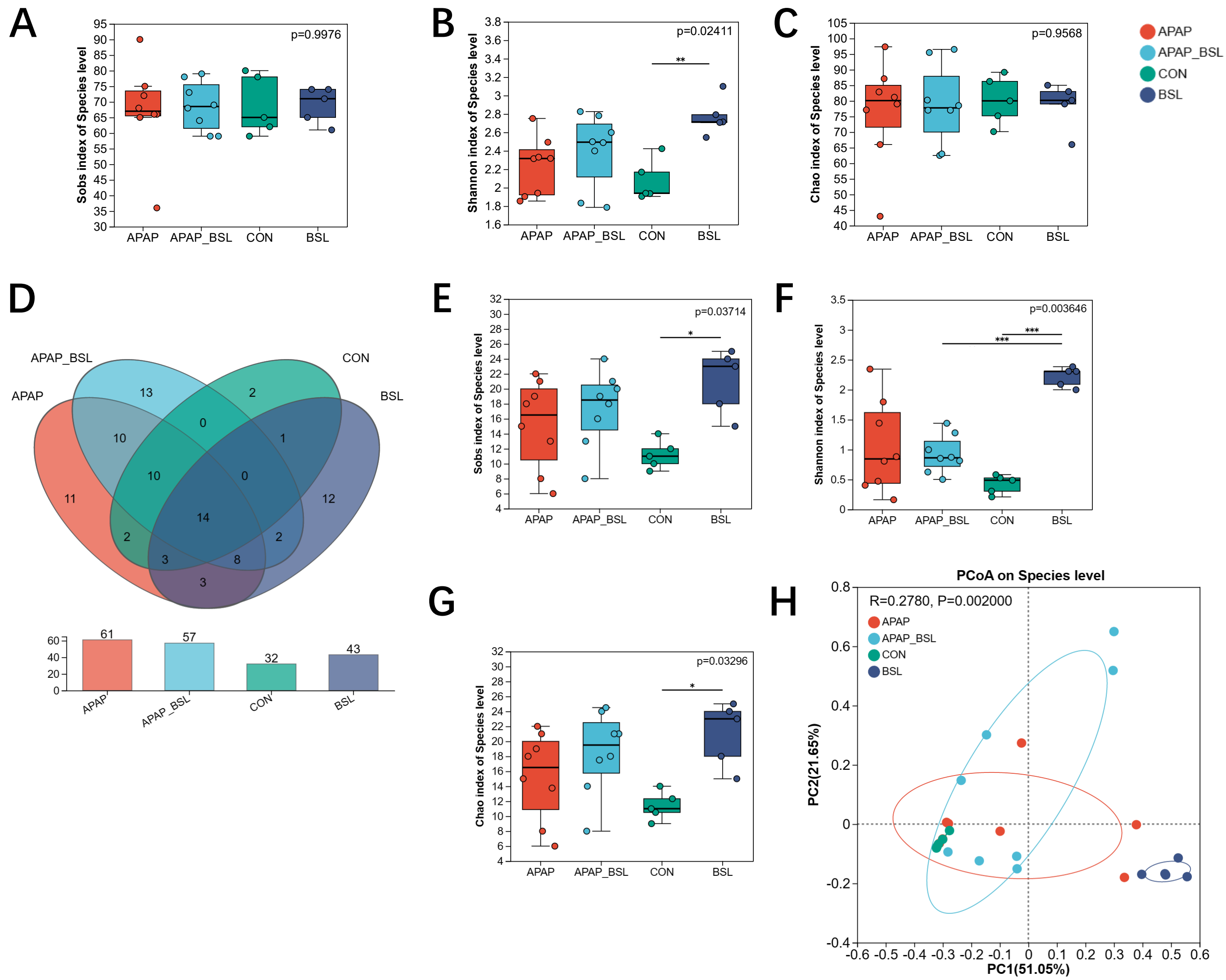

Supplementary Figure 5

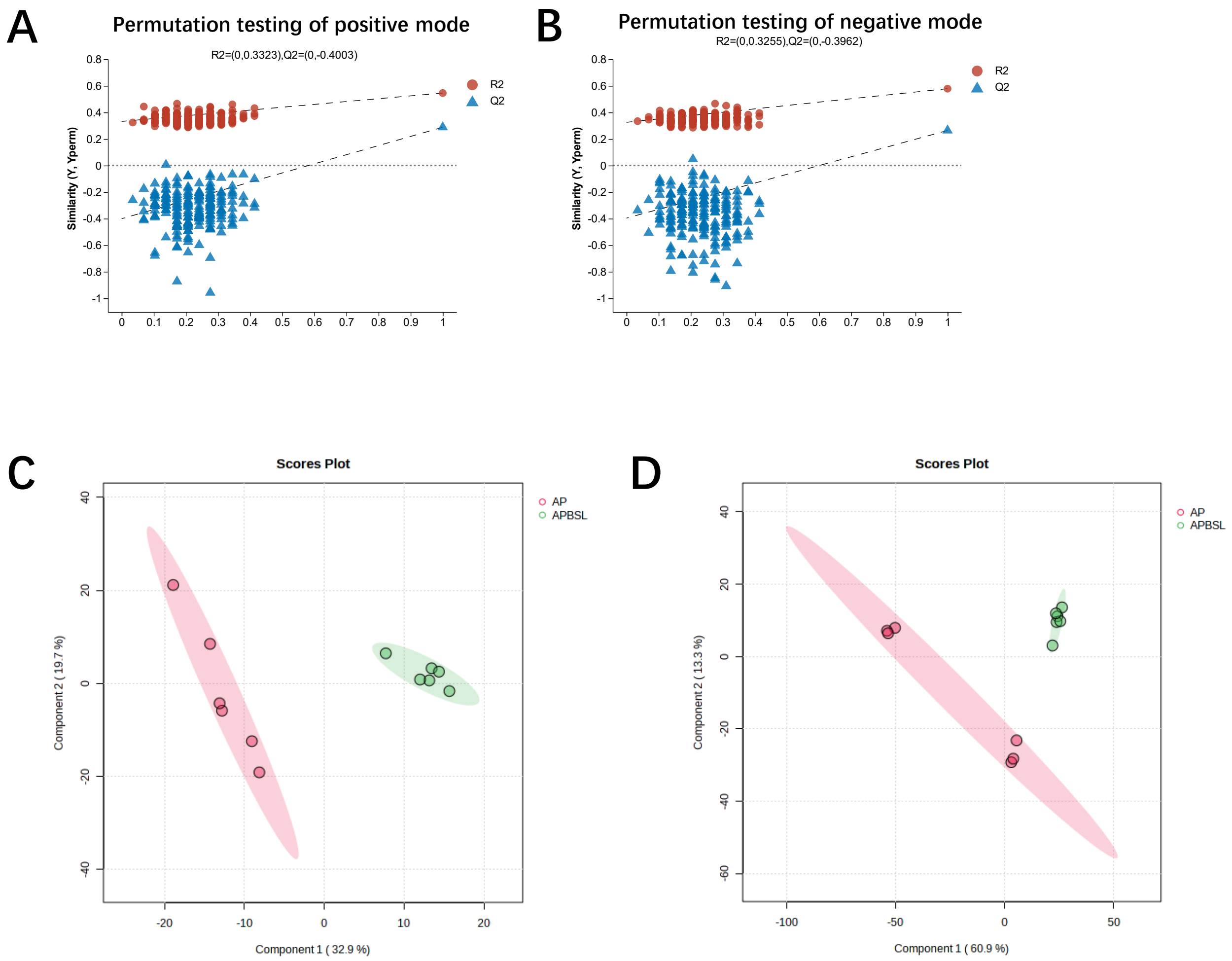

Supplementary Figure 6

A

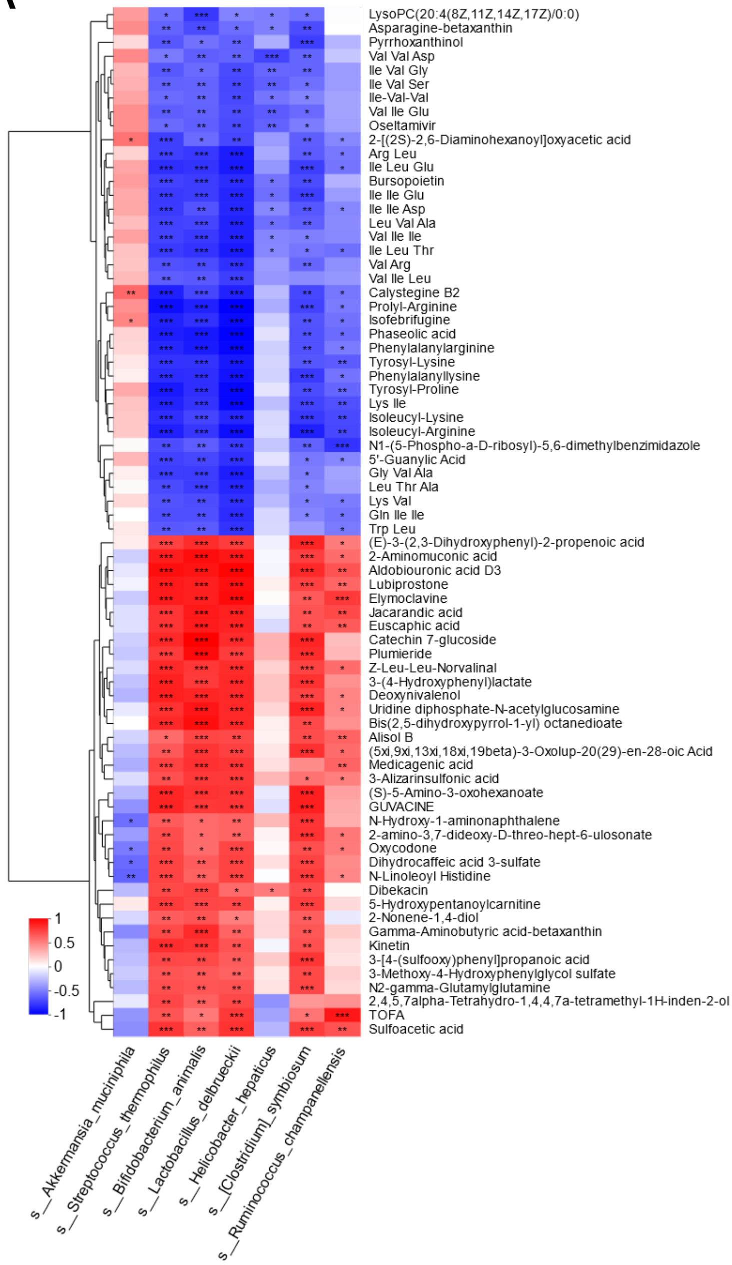

Supplementary Figure 7

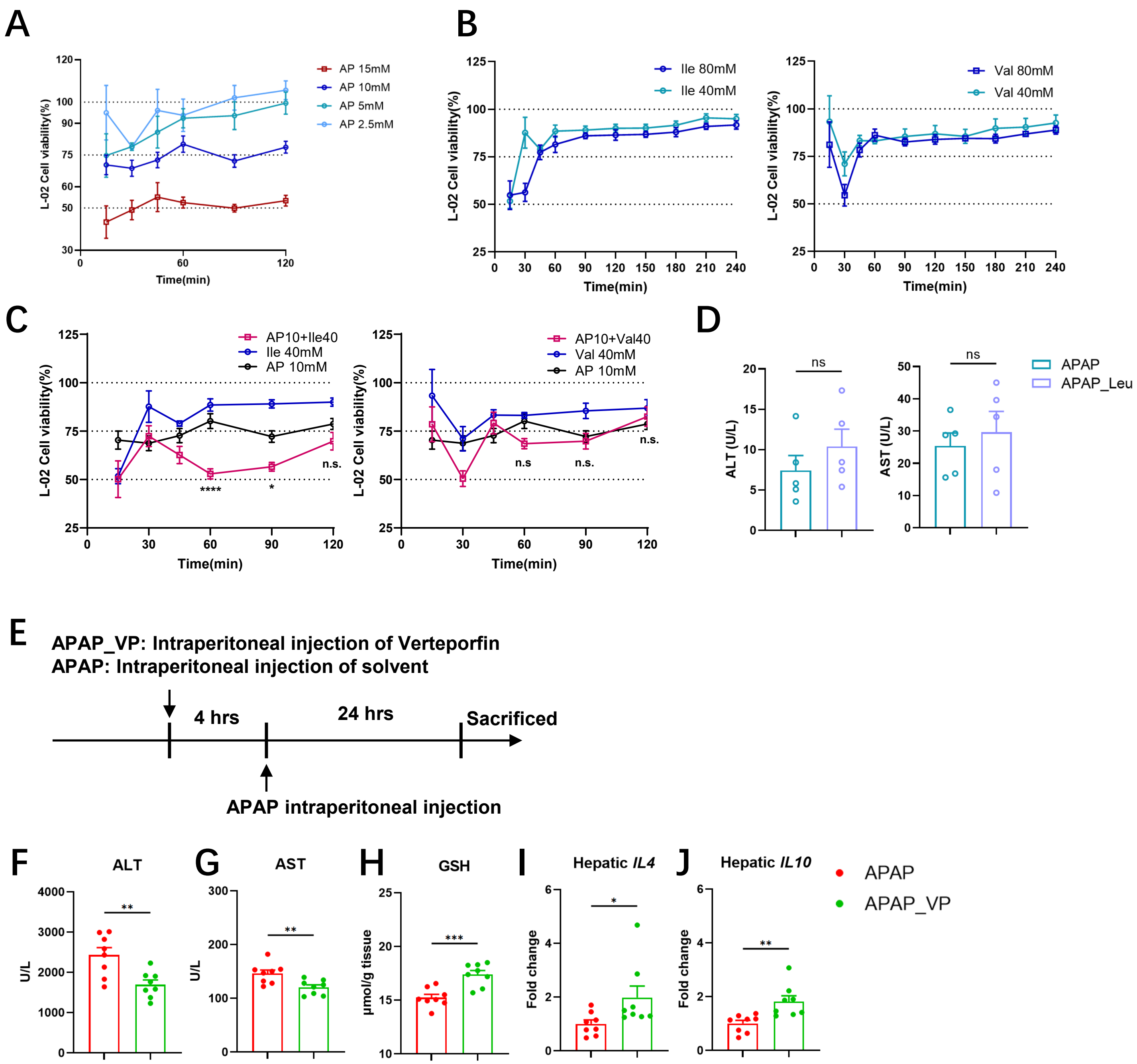

Supplementary Figure 8

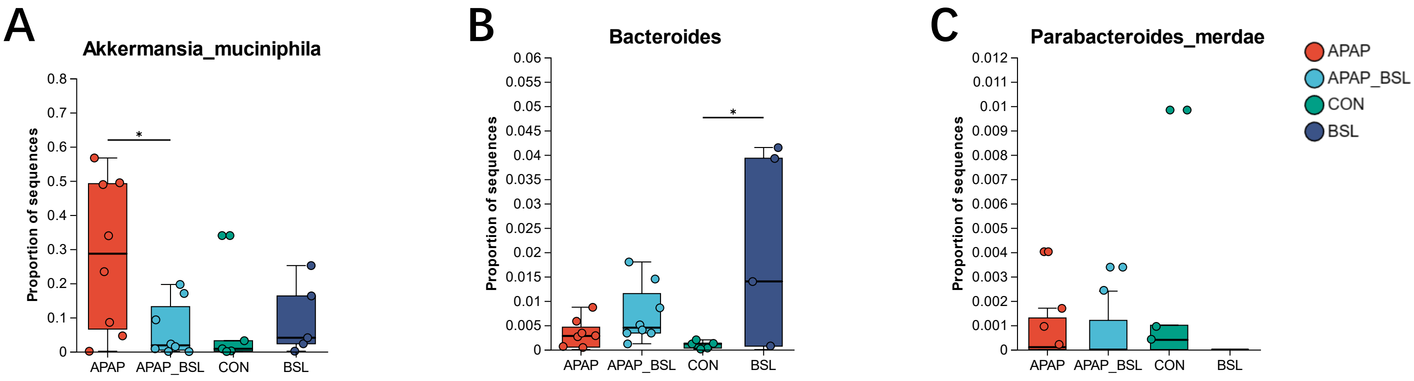

Supplement: Supplementary file 1 — Supplementary material 1. [file 13578_2025_1370_MOESM1_ESM.pdf]
