# Supplementary material for: Gram-positive probiotics improves acetaminophen-induced hepatotoxicity by inhibiting leucine and Hippo-YAP pathway
Source: Cell Biosci. 2025 Mar 7;15:32. doi: 10.1186/s13578-025-01370-5 (PMC11887100; doi:10.1186/s13578-025-01370-5)
Supplement: Supplementary file 2 — Supplementary material 2. [file 13578_2025_1370_MOESM2_ESM.docx]

**Supplementary Table 1 The demographic and laboratory characteristics of DILI patients**

|  | **DILI group**  **(n=16)** | **HC group**  **(n=18)** |
| --- | --- | --- |
| **Demographic parameter** |  |  |
| Age (years) | 48.5 (41.0-55.0) | 40.5 (36.3-57.8) |
| Male (%) | 6 (37.5%) | 5 (27.8%) |
| BMI (kg/m²) | 21.8 (19.8-24.0) | 21.82 (19.68-24.17) |
| **Laboratory parameter** |  | Reference interval |
| WBC (×10^12^/L) | 4.66 (4.28-4.97) | 3.5-9.5 |
| RBC (×10^12^/L) | 4.07 (3.89-4.59) | Male:4.3-5.8, Female: 3.8-5.1 |
| HB (g/L) | 127.0 (123.8-137.8) | Male:130-175, Female:115-150 |
| PLT (×10^9^/L) | 218.5 (186.5-253.5) | 125-350 |
| TB (μmol/L) | 53.5 (23.0-99.2) | 5.1-19.0 |
| DB (μmol/L) | 21.6 (11.5-61.8) | 1.7-6.8 |
| ALT (U/L) | 251 (109-515) | Male: 5-40, Female: 5-35 |
| AST (U/L) | 117 (80-300) | 8-40 |
| ALP (U/L) | 117 (95-183) | 40-150 |
| GGT (U/L) | 147 (121-183) | Male: 11-50, Female: 7-32 |
| Albumin (g/L) | 42.0 (38.3-43.3) | 35-55 |
| INR | 0.94 (0.92-0.98) | 0.80-1.20 |

Values are presented as median with interquartile range unless otherwise indicated. The laboratory parameters were not detected in HC group. WBC: white blood cell, RBC: red blood cell, HB: hemoglobin, PLT: platelet counts, TB: total bilirubin, DB: direct bilirubin, ALT: alanine aminotransferase, AST: aspartate aminotransferase, ALP: alkaline phosphatase, GGT: gamma-glutamyl transferase, INR: international normalized ratio.
