# Supplementary material for: Gram-positive probiotics improves acetaminophen-induced hepatotoxicity by inhibiting leucine and Hippo-YAP pathway
Source: Cell Biosci. 2025 Mar 7;15:32. doi: 10.1186/s13578-025-01370-5 (PMC11887100; doi:10.1186/s13578-025-01370-5)
Supplement: Supplementary file 5 — Supplementary material 5. [file 13578_2025_1370_MOESM5_ESM.docx]

**Supplementary Table 4**

| **Reagent or resource** | **Source** | **Identifier** |
| --- | --- | --- |
| **Antibodies** |  |  |
| Anti-rabbit CYP1A2 | Abcam | RRID: ab314666 |
| Anti-rabbit CYP2E1 | ABclonal | RRID: AB_2764178 |
| HRP conjugated Goat Anti-Rabbit IgG (H+L) | Servicebio | Cat# G1232 |
| **Chemicals** |  |  |
| Acetaminophen | Targetmol | Cat# T0065 |
| L-Leucine | Servicebio | Cat# GC304006 |
| L-Isoleucine | Servicebio | Cat# GC304005 |
| L-Valine | Servicebio | Cat# GC304017 |
| DMEM | Gibco | Cat# C11995500BT |
| PBS | Gibco | Cat# C10010500BT |
| Fetal bovine serum | Gibco | Cat# 10099141C |
| Penicillin/streptomycin | New cell & Molecular Biotech | Cat# C100C5 |
| CELLSAVING^TM^ | New cell & Molecular Biotech | Cat# C40100 |
| **Critical Commercial Assays** |  |  |
| ALT assay kit | Nanjing Jiancheng  Bioengineering Institute | Cat# C009-3-1 |
| AST assay kit | Nanjing Jiancheng  Bioengineering Institute | Cat# C010-2-1 |
| T-GSH/GSSG assay kit | Nanjing Jiancheng  Bioengineering Institute | Cat# A061-1-2 |
| MDA assay kit | Nanjing Jiancheng  Bioengineering Institute | Cat# A003-1-2 |
| SOD assay kit | Nanjing Jiancheng  Bioengineering Institute | Cat# A001-3-2 |
| Leucine ELISA lit | Jiangsu Meimian industrial Co., Ltd | Cat# MM-92615801 |
| Cell Counting kit-8 | HYCEZMBIO | Cat# HYCCK8 |
| RNA-easy isolation Reagent | Vazyme | Cat# R701-02 |
| HiScript III RT SuperMix for qPCR | Vazyme | Cat# R323-01 |
| ChamQ SYBR qPCR Master Mix | Vazyme | Cat# Q321-02 |
| **Experimental models: cell lines** |  |  |
| L-02 cells | This study | N/A |
|  |  |  |
| **Experimental Models: Organisms/Strains** |  |  |
| Specific Pathogen Free C57BL/6J | Beijing Weitong Lihua Experiment Animal Technology Co., Ltd. | N/A |
| **Bacterial strains** |  |  |
| *Bifidobacterium Longum* | Inner Mongolia Shuang Qi Pharmaceutical Co., Ltd. | NQ-1501 |
| *Streptococcus thermophilus* | Inner Mongolia Shuang Qi Pharmaceutical Co., Ltd. | NQ-5405 |
| *Lactobacillus delbrueckii subspecies bulgaricus* | Inner Mongolia Shuang Qi Pharmaceutical Co., Ltd. | NQ-2508 |
| **Primers for qPCR** | **Sequence (5' -> 3')** |  |
| Mouse *18S* | F: AGTCCCTGCCCTTTGTACACA  R: CGATCCGAGGGCCTCACTA | All primers were synthesized by Sangon Biotech (Shanghai) Co., Ltd. |
| Mouse *IL4* | F: GGTCTCAACCCCCAGCTAGT  R: GCCGATGATCTCTCTCAAGTGAT |  |
| Mouse *IL10* | F: GCTCTTACTGACTGGCATGAG  R: CGCAGCTCTAGGAGCATGTG |  |
| Mouse *IL1β* | F: TTCAGGCAGGCAGTATCACTC  R: GAAGGTCCACGGGAAAGACAC |  |
| Mouse *IL6* | F: CCAAGAGGTGAGTGCTTCCC  R: CTGTTGTTCAGACTCTCTCCCT |  |
| Mouse *TNFα* | F: GGTCTGGGCCATAGAACTGA  R: CAGCCTCTTCTCATTCCTGC |  |
| Mouse *Yap1* | F: ACCCTCGTTTTGCCATGAAC  R: TGTGCTGGGATTGATATTCCGTA |  |
| Mouse *Wwc1* | F: TGCTGAGGGAAACCAAAGCC  R: CTGGACCATAGGTCGGAGTG |  |
| Mouse *Lats1* | F: AAAGCCAGAAGGGTACAGACA  R: CCTCAGGGATTCTCGGATCTC |  |
| Mouse *Mst1* | F: AGCCCTCACGTAGTCAAGTAT  R: TCTTGTTCCGTAGCCGAATGATA |  |
| **Deposited data** |  |  |
| Full-length of 16S rRNA sequencing data | NCBI SRA Database | PRJNA1065322 |
| Full-length of ITS sequencing data | NCBI SRA Database | PRJNA1065322 |
| Transcriptome data | NCBI SRA Database | PRJNA1066211 |
| Non-targeted metabolome data | MetaboLights | MTBLS9447 |
