# Supplementary material for: Gram-positive probiotics improves acetaminophen-induced hepatotoxicity by inhibiting leucine and Hippo-YAP pathway
Source: Cell Biosci. 2025 Mar 7;15:32. doi: 10.1186/s13578-025-01370-5 (PMC11887100; doi:10.1186/s13578-025-01370-5)
Supplement: Supplementary file 6 — Supplementary material 6. [file 13578_2025_1370_MOESM6_ESM.docx]

**Supplementary Figure Legends**

**Supplementary Figure 1**

(A) The screening process of patients with DILI.

(B) The GMHI index between DILI group and HC group. ***p<0.001.

(C)-(E) Plot of correlation analysis between GMHI and indices of Alpha-diversity.

(F) Histogram of LDA analysis for DILI group and HC group. Microbes with LDA scores (log10) greater than 3 were considered differential microbiota.

(G) Cladogram of LEfSe analysis for DILI group and HC group. The red and blue nodes represented significantly enriched gut microbiota in DILI group and HC group, respectively. Yellowish nodes indicated microbiota without significant effect on intergroup differences. The inner to outer circles in the diagram denoted the classification level from phylum to species.

**Supplementary Figure 2**

(A) Daily body weight changes during the modeling period. n=6-7.

(B)-(C) ALT and AST plasma activities in mice with different single strain pretreatment. n=11-18.

(D) The level of hepatic GSH after 30 minutes of APAP injection (using Student’s t test). n=6.

(E) The protein expression of CYP2E1 after 30-minute of APAP administration. Scale bar=200 μm.

(F) The protein expression of CYP1A2 after 30-minute of APAP administration. Scale bar=500 μm.

One-way ANOVA was used to compare multiple groups if not otherwise specified. ns p>0.05, * p<0.05, ** p<0.01.

**Supplementary Figure 3**

(A)-(N) The Fpkm value of genes in the Hippo signaling pathway. n=5-8.

(O)-(Q) The Fpkm value of genes in the Nrf2 signaling pathway. n=5-8.

One-way ANOVA was used to compare multiple groups. * p<0.05, ** p<0.01, *** p<0.001, **** p<0.0001.

**Supplementary Figure 4**

(A)-(C) The indices of Sobs, Shannon, and Chao on bacterial species level among four groups (using Kruskal-Wallis rank sum test).

(D) Venn diagram of APAP, APAP_BSL, CON, and BSL group at the OTU level based on the full-length ITS sequencing. n=5-8.

(E)-(G) The indices of Sobs, Shannon, and Chao on fungal species level among four groups (using Kruskal-Wallis rank sum test).

(H) PcoA diagram of gut fungi at the species level (using bray-curtis distance algorithms, anosim analysis compared the difference). * p<0.05, ** p<0.01, *** p<0.001.

**Supplementary Figure 5**

(A)-(B) The validation plot of PLS-DA model of positive and negative mode, respectively. The upward trend of the regression line of Q2 and R2 indicated that the substitution test passed and the model was not overfitted.

(C)-(D) PLS-DA plot of hepatic metabolites from APAP group and APAP_BSL group in positive and negative mode, respectively. n=6.

**Supplementary Figure 6**

(A) Spearman’s correlation analysis between 7 bacteria and 74 metabolites. * p<0.05, ** p<0.01, *** p<0.001.

**Supplementary Figure 7**

(A) Effects of different concentrations of APAP on cell viability. n=6.

(B) Effects of different concentrations of isoleucine and valine on cell viability. n=6.

(C) Effects of APAP combined with isoleucine or valine on cell viability (using two-way ANOVA). The viability in cells added only with CCK8 was 100% (not shown in the figure). *indicated a significant difference between AP10+Ile40 and AP 10mM, and AP10+Val40 and AP 10mM. n=6.

(D) The serum levels of ALT and AST in APAP_Leu group and APAP group before APAP treatment. n=5.

(E) Experimental Scheme.

(F-G) The serum levels of ALT and AST in APAP_VP group and APAP group. n=8.

(H) The hepatic level of GSH in APAP_VP group and APAP group. n=8.

(I-J) Relative expression of anti-inflammatory factors (IL-4 and IL-10) in APAP_VP group and APAP group. n=8.

Student’s t test was used to compare two groups if not otherwise specified. n.s. p>0.05, * p<0.05, ** p<0.01, *** p<0.001

**Supplementary Figure 8**

(A)-(C) The proportion of *A. muciniphila*, *Bacteroides* and *Parabacteroides merdae* among four groups (using Kruskal-Wallis rank sum test). n=5-8. * p<0.05, ** p<0.01, *** p<0.001.
